# The Personalized Management of Primary Biliary Cholangitis in the Era of Precision Medicine: Current Challenges and Future Perspectives

**DOI:** 10.3390/jpm15120597

**Published:** 2025-12-03

**Authors:** Mario Romeo, Fiammetta Di Nardo, Claudio Basile, Carmine Napolitano, Paolo Vaia, Giuseppina Martinelli, Alessia De Gregorio, Luigi Di Puorto, Mattia Indipendente, Marcello Dallio, Alessandro Federico

**Affiliations:** Hepatogastroenterology Division, Department of Precision Medicine, University of Campania Luigi Vanvitelli, Piazza Miraglia 2, 80138 Naples, Italy; mario.romeo@unicampania.it (M.R.); fiammetta.dinardo@unicampania.it (F.D.N.); claudio.basile@unicampania.it (C.B.); carmine.napolitano1@studenti.unicampania.it (C.N.); paolo.vaia@studenti.unicampania.it (P.V.); giuseppina.martinelli@studenti.unicampania.it (G.M.); alessia.degregorio2@studenti.unicampania.it (A.D.G.); luigi.dipuorto2@studenti.unicampania.it (L.D.P.); mattia.indipendente@studenti.unicampania.it (M.I.); alessandro.federico@unicampania.it (A.F.)

**Keywords:** personalized medicine, cholestasis, autoimmune, fibrosis, biliary tract disease

## Abstract

Primary biliary cholangitis (PBC) is a heterogeneous autoimmune liver disease in which clinical presentation, disease progression, and response to therapy vary markedly from patient to patient. This heterogeneity reflects its complex, multifactorial, and not-completely elucidated pathogenesis. Currently, serological markers are available to non-invasively diagnose PBC, reserving liver biopsy for selected cases with atypical presentations or diagnostic uncertainty. Anyway, the accurate non-invasive prediction of liver-related and non-liver-related (i.e., extra-hepatic, including pruritus) outcomes remains an open challenge, as well as an urgent need, considering the great variability in clinical course and prognosis reported in PBC patients. Moreover, although ursodeoxycholic acid (UDCA) remains the standard first-line treatment, not all individuals respond equally, either in terms of therapeutic efficacy or timing of biochemical improvement. This further variability in treatment response underscores the inadequacy of uniform management approaches and reinforces the urgent need for personalized medicine, where treatment decisions are guided by patient-specific biological and clinical parameters. In this scenario, the identification and validation of non-invasive predictive biomarkers capable of detecting early therapeutic responsiveness are pivotal for optimizing care pathways. Finally, a growing portion of patients show an insufficient UDCA response or are UDCA intolerant, making the identification of novel strategies of care an urgent need. Concerning this, very recently, new therapeutic options beyond UDCA targeting, among the other pathways, bile acid metabolism (including the modern Peroxisome Proliferator-Activated Receptor agonists), immune regulation, and fibrogenesis, have expanded the treatment landscape. In the Era of Precision Medicine, these diagnostic, prognostic, and therapeutic innovations, by reflecting the complexity of PBC pathogenesis, underline the cruciality of a patient-tailored strategy to improve outcomes and mitigate disease progression. The present review reports recent advances, highlights ongoing challenges, and outlines future perspectives in the management of PBC.

## 1. Background

### 1.1. Primary Biliary Cholangitis: When the Disease Definition Can Illustrate the Etiopathogenesis

Primary biliary cholangitis (PBC) is a chronic inflammatory cholestatic autoimmune liver disease, characterized by chronic non-suppurative phlogosis, selectively targeting the small intrahepatic bile ducts, ultimately leading to chronic cholestasis, progressive fibrosis, and cirrhosis in advanced stages [1].

Despite its connotation of rare liver disease [1], PBC affects individuals of all sexes, races, and ethnicities, with a reported prevalence ranging from 19 to 402 cases per million population and an incidence between 0.33 and 5.8 cases per million per year across Europe, North America, Asia, and Australia [2]. Recent epidemiological data demonstrate that the incidence of PBC is highest in North America (2.75 per 100,000 persons), followed by Europe (1.86 per 100,000 persons), and lowest in the Asia-Pacific region (0.84 per 100,000 persons) [2]. A key epidemiological feature is the marked female predominance. Historically, female-to-male ratios as high as 10:1 were reported; however, more recent studies have shown that while PBC remains more common in women, the male-to-female distribution is closer to 1:4–1:6. This is clinically relevant, as PBC in men is associated with worse outcomes, including a lower likelihood of biochemical response to the first therapeutic line and a higher risk of progression to cirrhosis and hepatocellular carcinoma (HCC) [3].

Although the precise etiology remains elusive, twin studies have demonstrated concordance rates as high as 63% in monozygotic pairs, strongly supporting a genetic contribution [4]. Genome-wide association studies (GWAS) have further identified HLA allelic variants, particularly HLA-DRB108, as conferring increased risk, whereas HLA-DRB111 and HLA-DRB113 appear protective [5,6]. Beyond the HLA locus, several non-HLA risk loci—including IL12A and IL12RB2—underscore the central role of IL-12–driven Th1 responses in sustaining autoimmune injury [7].

Relevantly, several epigenetic modifications provide an additional mechanistic link between inherited risk and environmental triggers.

Among these, aberrant DNA methylation, histone modifications, and microRNA (miR-) dysregulation have all been implicated in disease progression. Notably, miR-506 overexpression has been shown to impair cholangiocytes’ physiology, while miR-34a promotes fibrogenesis through activation of the Transforming Growth Factor Beta 1 (TGF-β1)/SMAD signaling pathway [8,9]. On the other hand, various environmental factors may act as pivotal disease triggers in predisposed hosts. Recurrent Escherichia coli infections have been proposed to drive autoimmunity via molecular mimicry against the pyruvate dehydrogenase complex E2 subunit (PDC-E2), the immunodominant autoantigen in PBC [10,11]. Similarly, xenobiotics such as 2-octynoic acid have been implicated in loss of immune tolerance and can reproduce PBC-like features in murine models [12]. Interestingly, cigarette smoking has been consistently associated with both increased risk of PBC and faster progression to advanced fibrosis [13,14].

Taken together, the pathogenetic framework of PBC posits that, in genetically predisposed individuals with epigenetic dysregulation, exposure to environmental triggers initiates the breakdown of immune tolerance. This leads to both humoral and cellular responses against cholangiocytes [15,16], characterized by production of antimitochondrial antibodies (AMAs)—targeting the abovementioned PDC-E2—periportal lymphocytic infiltration, and activation of CD4^+^ and CD8^+^ T cells [17]. The resulting cholangiocyte apoptosis and disruption of the protective bicarbonate umbrella facilitate hydrophobic bile acid accumulation, thereby perpetuating epithelial injury and chronic inflammation, ultimately culminating in the hallmark lesion of nonsuppurative destructive cholangitis [18,19].

### 1.2. Primary Biliary Cholangitis Diagnosis: Redefining the Current Role of Liver Biopsy

Consistent with the nature of chronic cholestatic and autoimmune disease, the biochemical signature of PBC includes persistent elevation of alkaline phosphatase (ALP), γ-glutamyltransferase (GGT), and bilirubin serum levels, frequently accompanied by increased immunoglobulin M (IgM) levels [1,20].

Currently, the diagnosis of PBC relies on the fulfilment of at least two of the following three key criteria: chronic biochemical cholestasis, the presence of autoantibodies AMA or disease-specific antinuclear antibodies (ANAs), and compatible histological features (nonsuppurative destructive cholangitis and interlobular bile duct loss) [20,21,22]. Clinical suspicion typically arises in a patient—most often a middle-aged woman—presenting with persistent cholestatic liver enzyme abnormalities, particularly an elevation of ALP ≥ 1.5 times the upper limit of normal (ULN) for more than six months [20,23].

Non-invasive imaging, including abdominal ultrasound and magnetic resonance cholangiopancreatography (MRCP), is essential to exclude extrahepatic obstructive cholangiopathies [24,25]. In the absence of alternative causes, a diagnosis of PBC can be established without liver biopsy in patients presenting with a cholestatic biochemical profile and either AMA positivity or disease-specific ANA (anti-sp100, anti-gp210) at titers > 1:40. AMA testing—typically the M2 subtype (AMA-M2)—remains the diagnostic cornerstone, with pooled sensitivity and specificity of 84.5% and 97.8%, respectively, as demonstrated in a recent meta-analysis of 24 studies [26]. Disease-specific ANA are detected in approximately 50% of patients and are particularly relevant in AMA-negative cases; they also carry prognostic value, being associated with accelerated disease progression [27,28]. In AMA-negative patients, testing for disease-specific ANA (anti-sp100, anti-gp210) is crucial to establish the diagnosis of “AMA-negative PBC” [20].

Despite the biochemical variable recognized by clinical practice guidelines (CPGs) as a cornerstone to serologically define cholestasis is ALP (and not GGT) [20], a revolutionary report by the Swiss PBC Cohort Study Group revealed a non-negligible proportion of AMA-M2 positive patients with normal ALP levels presenting with a histologically proven PBC [29]. For the first time, the authors show histological diagnosis of PBC in AMA-negative/PBC-specific ANA-positive subjects and the potential role of GGT as a biomarker in PBC patients with normal baseline ALP levels [29].

Altogether, these emerging findings highlight the need to revise and update the current CPGs for the diagnosis of PBC [20,30,31], considering the incomplete coverage of the present recommendations of the whole extent of PBC presentation, with important clinical implications in terms of personalized management and timely treatment initiation. In line with this, a relevant proportion of patients with early-stage PBC have been subsequently shown to typically present a discordant GGT and ALP levels pattern, showing more pronounced GGT elevations and normal ALP levels in a non-negligible subset of individuals [32]. Moreover, in cases of AMA negativity, the presence of ANA—particularly centromere pattern positivity—may support the suspicion of early PBC [32]. Clinically, markedly elevated GGT levels, even in the context of normal ALP, combined with ANA positivity (especially centromere pattern) in the absence of AMA, should prompt consideration of early-stage PBC [32]. In such settings, or in the case of high clinical suspicion, liver biopsy may be required to confirm the histological pattern of nonsuppurative destructive cholangitis with interlobular bile duct injury [33]. Despite their high diagnostic accuracy, these serological markers are not absolute; therefore, biopsy remains the gold standard in uncertain or atypical presentations [34].

Liver biopsy is generally unnecessary in typical cases—defined by persistent cholestasis, AMA or specific ANA positivity, and absence of competing etiologies—where diagnosis can be confidently made without histology [25].

However, biopsy remains indispensable in selected scenarios: (1) AMA-negative or ANA non-specific cases requiring histological confirmation of biliary injury [31]; (2) suspected autoimmune hepatitis (AIH) overlap, particularly when ALP/alanine aminotransferase (ALT) < 1.5, serum IgG is elevated, and anti-smooth muscle antibodies are present, where biopsy documents interface hepatitis and bridging necrosis in addition to biliary lesions [20,31]; (3) atypical presentations (e.g., disproportionately high aminotransferases, rapid deterioration, suspected infiltrative disease); (4) fibrosis staging and prognostic assessment, using systems such as Ludwig classification [20,31].

Nevertheless, biopsy is limited by sampling variability, procedure-related risks (notably bleeding), and the increasing availability of noninvasive prognostic tools such as the GLOBE score and the UK-PBC risk score. Therefore, accordingly, current CPGs recommend reserving liver biopsy for cases with diagnostic uncertainty or mixed phenotypes [20,35].

**Figure 1:**
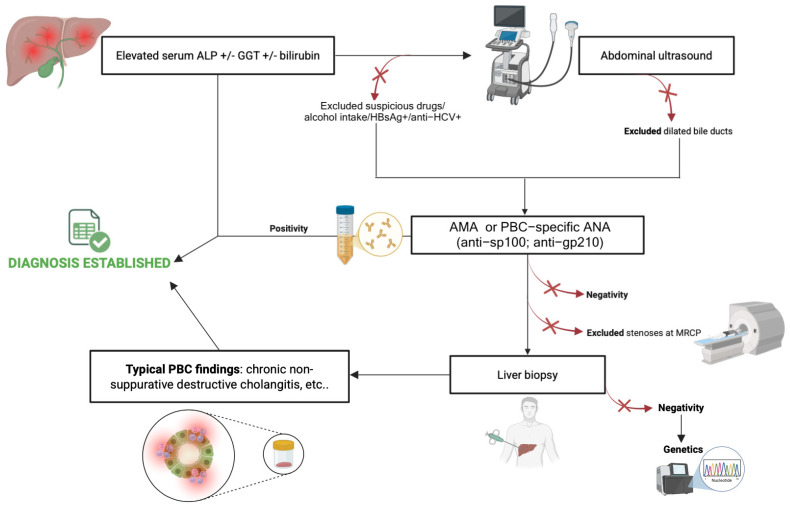
The current diagnostic decision process in the cholestasis scenario: biochemical, serological, and histological features to establish PBC diagnosis. ALP: alkaline phosphatase; GGT: gamma-glutamyltransferase; HBsAg: Hepatitis B virus surface antigen; HCV: Hepatitis C virus; AMA: antimitochondrial autoantibodies; ANA: antinuclear autoantibodies.

Figure 1 summarizes the current approach to properly manage patients presenting with cholestasis and achieving the PBC diagnosis in routine clinical practice (Figure 1).

### 1.3. Personalizing the Management Strategies for Primary Biliary Cholangitis: An Urgent Need

Despite its connotation of rare disease [1], PBC imposes a significant burden in terms of chronic morbidity, reduced quality of life (QoL), and long-term healthcare utilization. Importantly, PBC remains one of the leading causes of cirrhosis and liver transplantation worldwide [36,37]. Beyond its histological and biochemical progression, PBC exerts a profound negative impact on health-related quality of life (HRQoL), with extrahepatic manifestations, including intractable fatigue, pruritus, and osteosarcopenia, significantly impairing daily functioning, often even in patients achieving biochemical response to therapy [38,39]. Moreover, fatigue, pruritus, and osteosarcopenia—often present from early stages [40]—contribute to psychosocial distress and functional impairment [41]. Furthermore, delayed PBC diagnosis or suboptimal response to first-line therapy may accelerate progression to cirrhosis, necessitating advanced interventions, including transplantation.

In the current era of Precision Medicine, the need for a tailored therapeutic approach has become increasingly evident, particularly in chronic progressive liver disorders where clinical heterogeneity and treatment response variability require individualized strategies [42]. These features appear even more critical in the PBC setting, where despite the progressive designation of several molecules and the emergence of new medications, the large interindividual variability of hepatic (and extrahepatic) manifestations, both in terms of the time of presentation, disease severity, and therapeutic response, imposes the optimization of models accurately predicting the various clinical outcomes. In this sense, accurate prediction of PBC’s course and prognosis is essential for determining the risk throughout the disease continuum, enabling stratified follow-up care, and ideally allowing personalized therapies.

Following the introduction of ursodeoxycholic acid (UDCA) as first-line therapy in the late 1990s, the “treatment response” emerged as a strong predictor of long-term outcome in PBC, nowadays effectively representing the cornerstone of prognosis definition in this setting [43]. Since then, several criteria to define UDCA response have been proposed, by variably combining biochemical variables and testing different thresholds, aiming to identify incomplete response to UDCA, the most robust predictor of long-term prognosis, and thereby identifying patients that are at risk of liver-related complications. Despite most models evaluating treatment response at 12 months, recent research suggests that evaluating biochemical response at 6 months may offer equivalent predictive performance to 12-month assessments, facilitating earlier identification of patients who might benefit from second-line therapies [44,45].

Early approaches relied on dichotomous response criteria, which classify patients as responders or non-responders based on predefined biochemical thresholds. These scores are simple and clinically intuitive, but the intrinsic binary stratification inevitably leads to a loss of information and does not reflect the continuous nature of risk. More recently, continuous risk models have been introduced: by incorporating multiple biochemical and demographic variables, these criteria provide individualized time-related estimates of transplant-free survival and have demonstrated superior prognostic performance compared to dichotomous definitions [43].

Together, these advances illustrate the evolving landscape of prognostic modelling in PBC, where the challenge remains to balance accuracy, non-invasive approaches, and clinical applicability while moving toward earlier and more personalized therapeutic decision-making.

Considering this background, this review aims to critically appraise recent therapeutic advances, present prognostic and therapeutic response predictive (invasive and noninvasive) tools, ultimately highlighting ongoing challenges, and outlining future perspectives in the management of PBC.

## 2. Therapeutic Opportunities to Treat Patients with Primary Biliary Cholangitis: Today’s Certainties and Tomorrow’s Challenges

Current therapeutic strategies in PBC are designed to achieve multiple clinical endpoints [46]. First, biochemical response, particularly normalization of ALP and bilirubin serum levels, is currently considered a key surrogate marker for improved prognosis [46]. Second, histologic stabilization or regression—though less frequently assessed in routine practice—remains a critical objective, especially in early-stage disease [20,30,46]. In addition, prevention of cirrhosis and its complications, including portal hypertension and hepatic decompensation, is central to long-term management [20,30]. Moreover, risk reduction for primary liver cancer is increasingly recognized, particularly in patients with advanced fibrosis or suboptimal response to the first-line therapy [20,30]. Finally, improvement in health-related QoL—addressing symptoms such as fatigue and pruritus—is essential, given the substantial impact on daily functioning and psychosocial status [47].

**Figure 2:**
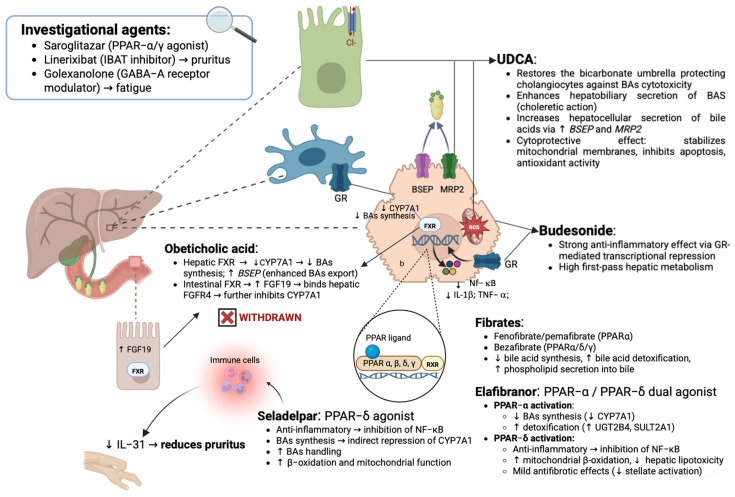
The current therapeutic targets, along with their related pathogenetic mechanisms and downstream pathways, in PBC. UDCA: Ursodeoxycholic acid; PPAR: Peroxisome Proliferator-Activated Receptors; BAS: bile acids; IBAT: ileal bile acid transporter; BSEP: Bile Salt Export Pump; MR2: Multidrug resistance protein 2; FXR: farnesoid X receptor; FGFR-4: Fibroblast growth factor receptor 4; IL: Interleukin; TNF: Tumor Necrosis Factor; GR: glucocorticoid receptor; NF-kB: nuclear factor kappa B; UGT: uridine diphosphate glucuronosyltransferases; CYP: Cytochromes P; SULT2A1: Bile salt sulfotransferase; ↑: increased; ↓: decreased.

Altogether, these goals guide the use of first-line therapy with UDCA and second-line therapy in selected patients, with ongoing research refining the therapeutic targets and expanding the options for disease-modifying interventions. Figure 2 summarizes the main targets with related mechanisms and regulated downstream pathways, reporting the available and under investigation molecules in the management of PBC (Figure 2).

### 2.1. Ursodeoxycholic Acid (UDCA): The Mainstay of Therapy in Primary Biliary Cholangitis

Despite several novel emerging drugs, UDCA remains the universally recommended first-line therapy for patients receiving a diagnosis of PBC.

This molecule represents the 7β-epimer of chenodeoxycholic acid, a naturally occurring bile acid that constitutes only a minor fraction of the human bile acid pool [48]. In line with this, the therapeutic rationale stems from the replacement of endogenous hydrophobic, cytotoxic bile acids with the more hydrophilic and less injurious UDCA, thereby mitigating bile acid-induced liver injury [48].

Anyway, the hepatoprotective effects of UDCA appear multifaceted, supporting the pleiotropic mode of action of this drug: by enhancing hepatocellular and cholangiocellular secretion, UDCA reduces cholestasis [49], while stabilizing hepatocyte membranes and counteracting bile acid- and cytokine-induced cytotoxicity [50]. Furthermore, UDCA has been demonstrated to exert intrinsic anti-inflammatory and immunomodulatory effects through stimulation of glutathione synthesis, activation of the transcription factor Nuclear Factor Erythroid 2–Related Factor 2 (NRF2), and induction of downstream antioxidant genes, thereby enhancing resistance to oxidative stress [51].

The optimal currently proposed UDCA dose is 13–15 mg/kg/day, administered either as a single daily dose or in divided doses to improve tolerability, and treatment is continued lifelong [20,52]. In this setting, randomized controlled trials first demonstrated that UDCA significantly improves cholestatic biochemical parameters, particularly ALP and bilirubin, and slows histological progression, with the greatest benefit when initiated in early disease [53,54]. Subsequently, long-term research further demonstrated that UDCA not only improves biochemical markers but also prolongs transplant-free survival and delays cirrhosis-related complications [55].

More recently, the Global PBC Study Group confirmed in a multicenter cohort of 3902 patients (median follow-up 7.8 years) that UDCA significantly improves transplant-free survival, with a 10-year probability of 79.7% in treated patients versus 60.7% in untreated patients (HR 0.46). Notably, the survival benefit persisted even among patients with suboptimal biochemical response (HR 0.56), reinforcing its role as a universal first-line therapy irrespective of baseline disease stage or initial treatment response [56].

Beyond the efficacy-related features, the safe profile of UDCA represents another critical element enhancing the generalizability of the administration as first-line therapy in the PBC setting.

Indeed, UDCA is generally safe and well-tolerated, and the mainly reported adverse effects, including diarrhea, flatulence, weight gain, and exacerbation of pruritus, occur in a minority of patients, are usually mild, and rarely necessitate discontinuation [57].

Nevertheless, certain limitations remain. Approximately one-third of patients fail to achieve an adequate biochemical response, defined by normalization or reduction (<1.67 × ULN) of ALP within one year [58,59]. These nonresponders retain a significantly increased risk of liver failure, transplantation, or death despite therapy. In addition, UDCA has little impact on PBC-related symptoms such as pruritus and fatigue, which frequently persist even in responders.

Therefore, while UDCA represents the therapeutic cornerstone of PBC with well-established efficacy and prognostic benefit, its incomplete efficacy and lack of symptomatic relief highlight the need for second-line and adjunctive therapies.

### 2.2. Beyond Ursodeoxycholic Acid: Lights, Shadows, and Promises of Second-Line Therapies in PBC

The introduction and availability of UDCA as first-line therapy for PBC significantly reduced disease progression to cirrhosis and decreased the number of liver transplants required for this condition [60].

Nevertheless, approximately 30–40% of patients show an inadequate biochemical response and remain at risk of developing advanced disease stages, including cirrhosis [61,62]. In these patients, UDCA therapy should be continued and complemented by second-line treatments. The identification of candidates for add-on therapy is best guided by validated prognostic tools such as the GLOBE score and the UK-PBC score, which allow for accurate risk stratification and early identification of patients with poor prognosis [63,64].

#### 2.2.1. Role of Budesonide in the Treatment of PBC and Relative Limitations

Budesonide, a synthetic corticosteroid with high hepatic first-pass metabolism and reduced systemic toxicity compared to conventional glucocorticoids, has initially been investigated as adjunctive therapy (i.e., as UDCA add-on treatment) in PBC [30].

Early randomized controlled studies suggested that budesonide in combination with UDCA improved biochemical and histological outcomes compared with UDCA alone [65,66]. However, altered pharmacokinetics in cirrhotic patients can result in deleterious effects, including portal vein thrombosis, and thus its use is contraindicated in cirrhosis [67,68].

Nonetheless, a more recent randomized controlled trial found that although budesonide plus UDCA improved biochemical parameters, it failed to achieve histological benefit or reduce fibrosis progression compared to UDCA monotherapy [69]. Thus, while combination therapy may be advantageous in selected non-cirrhotic patients, its impact on long-term clinical outcomes, including mortality and transplant-free survival, remains uncertain. Finally, despite the autoimmune pathogenesis of PBC, corticosteroids and immunosuppressive agents have shown limited efficacy, with the notable exception of PBC-autoimmune hepatitis overlap syndrome, where immunosuppression remains essential.

These unmet needs have driven therapeutic innovation over the past decade. In this sense, the conditional approval of obeticholic acid (OCA) by the U.S. Food and Drug Administration (FDA) and the European Medicines Agency (EMA) in 2016 represented a major milestone [70].

#### 2.2.2. Obeticholic Acid: Initial Enthusiasm Culminated in a Cruel Twist and Unfortunate Fate

OCA, a semisynthetic derivative of chenodeoxycholic acid, acts as a potent farnesoid X receptor (FXR) agonist, a nuclear receptor critically involved in the regulation of bile acid synthesis, transport, and enterohepatic circulation [70,71,72,73].

The pivotal phase III POISE trial (NCT03505723) initially demonstrated that OCA, either as monotherapy or in combination (i.e., add-on therapy) with UDCA, achieved significant biochemical improvement in patients with an inadequate response or intolerance to UDCA [74]. In POISE, 46–47% of patients in the OCA arms reached the composite primary endpoint, compared with 10% in the placebo group. Moreover, OCA reduced prognostic risk scores (GLOBE and UK-PBC), suggesting an improvement in transplant-free survival [74,75,76]. However, pruritus was the most frequent adverse event, affecting up to 68–77% of patients depending on treatment duration, and was dose-dependent [76,77].

In 2021, the FDA restricted OCA use in patients with decompensated cirrhosis, prior decompensation events, or compensated cirrhosis with evidence of portal hypertension, due to safety concerns [78]. Subsequently, the confirmatory COBALT trial (NCT02308111) failed to demonstrate a significant benefit of OCA on hard clinical endpoints (death, transplant, hepatic decompensation, MELD ≥ 15), with nearly identical event rates between OCA and placebo (28.6% vs. 28.9%; HR 1.01) [79].

Anyway, this trial has drawn scrutiny for several methodological shortcomings that may undermine the interpretability and regulatory strength of its findings. Firstly, rather than employing a fully randomized placebo-controlled design, COBALT incorporated a pre-specified external control arm derived from the Komodo Health U.S. claims database. While this approach aimed to reflect real-world comparators, it introduces substantial risk of selection bias, unmeasured confounding, and data heterogeneity. The absence of contemporaneous randomization limits the ability to attribute observed differences in clinical outcomes solely to the intervention [79,80]. Moreover, the use of external controls inherently precludes blinding and allocation concealment, both of which are critical safeguards against performance and detection bias. This design choice may have influenced clinical decision-making or outcome assessment in the OCA-treated cohort [79,80].

Despite these relevant criticisms, as a result of the failure of this confirmatory trial, in June 2024, the EMA recommended withdrawal of OCA’s conditional approval, concluding that its benefits no longer outweighed its risks [80]. This decision ultimately led to the unavailability to continue to adopt this drug in several European countries, creating a black hole in the treatment of PBC patients, particularly in UDCA-intolerant individuals.

While the withdrawal of conditional approval by the EMA reflects the lack of confirmatory evidence on hard clinical endpoints, it is important to acknowledge that certain patient subgroups may still theoretically derive benefit. Specifically, data from the POISE trial suggested that patients with an inadequate biochemical response to UDCA, but without advanced cirrhosis or portal hypertension, achieved significant improvements in surrogate markers and prognostic scores [74]. Moreover, UDCA-intolerant individuals represented a population for whom OCA offered a therapeutic alternative, albeit limited by pruritus and safety concerns [76,77]. Although these observations cannot override the regulatory decision, they highlight that OCA’s pharmacological activity as a potent FXR agonist may retain clinical relevance in carefully selected, lower-risk patients.

Anyway, this regulatory decision marked a critical turning point in the PBC management, also emphasizing the limitations of relying on surrogate biochemical markers alone [80]. In this sense, the setback of OCA reinforced the urgent need for more robust, long-term randomized controlled trials in PBC, which, anyway, continues to represent a challenge given the epidemiological representation of the disease and its slow natural history.

Meanwhile, in this disrupted scenario, novel therapeutic classes—most notably peroxisome proliferator-activated receptor (PPAR) agonists—have been recently and progressively emerging as promising second-line strategies.

#### 2.2.3. Peroxisome Proliferator-Activated Receptors as a Crucial Target for PBC Therapy

Peroxisome proliferator-activated receptors (PPARs) are fatty acid-activated transcription factors of the nuclear hormone receptor superfamily that regulate energy metabolism. Currently, three PPAR subtypes have been identified: PPARα, PPARγ, and PPARβ/δ [81].

PPAR activation is mechanistically linked to the regulation of bile acid synthesis, metabolism, and transport. In particular, PPARs represent a family of intracellular nuclear receptors that are activated by elevated lipid concentrations.

These receptors regulate lipid metabolism, storage, intracellular trafficking, and elimination, thereby influencing multiple pathways relevant to cholestasis [82].

In light of this, PPAR agonists have emerged as promising therapeutic alternatives for PBC, with the potential to improve biochemical responses, reduce mortality, and alleviate pruritus [83].

##### Fibrates: Mechanisms and Indications of “Classic” PPAR Agonists in PBC

Fibrates have been extensively investigated as therapeutic agents for cholestatic liver diseases, given their potential to reduce bile acid synthesis and mitigate bile acid-mediated hepatic inflammation [82]. Currently, in the generic clinical practice, three molecules are approved for use: fenofibrate (target: PPAR-α), pemafibrate (target: PPAR-α), and bezafibrate (targets: PPAR-α, β/δ, γ) [84].

Fenofibrate and pemafibrate are selective PPARα agonists regulating cholesterol and bile acid homeostasis by inhibiting the enzymatic activity of cholesterol 7α-hydroxylase (CYP7A1), leading to reduced bile acid synthesis, while also enhancing bile acid detoxification and promoting phospholipid export. Moreover, fenofibrate reduces hepatic inflammation by inhibiting the nuclear factor κB pathway [85,86].

Bezafibrate acts as a pan-PPAR agonist, thereby exerting broader biological effects. In particular, PPARγ activation contributes to lipoprotein modulation and mediates the anti-inflammatory and antifibrotic actions of bezafibrate [87].

Looking specifically to the PBC setting, cumulating reports of using fenofibrate (a synthetic PPAR-alpha agonist) and bezafibrate (a pan-PPAR agonist) for the adjunctive treatment of PBC, including a variety of open-label experiences and, more recently, also randomized controlled data (for bezafibrate) [88,89,90], have progressively proposed these molecules as potential alternatives in patients presenting with inadequate response to UDCA.

Two decades ago, the first evaluation of bezafibrate in PBC initially demonstrated normalization of ALP and significant reductions in IgM levels in patients with precirrhotic disease [91]. More recently, robust evidence has been provided by the BEZURSO randomized controlled trial (NCT01654731), which included 100 PBC patients with inadequate response to UDCA [90]. Bezafibrate (dosage 400 mg/day) was associated with normalization of ALP in 67% of treated patients versus 2% in the placebo arm, although mild increases in creatinine and myalgia were reported [90]. Complementary real-world evidence from a large retrospective cohort database in Japan, encompassing 3908 patients with PBC, has demonstrated the potential survival benefit of bezafibrate when added to UDCA [92]. In this study, using propensity score matching, Tanaka et al. showed that combination therapy with UDCA and bezafibrate was associated with a significantly reduced risk of all-cause mortality or liver transplantation (adjusted hazard ratio [HR] 0.33), as well as liver-related death or transplantation (adjusted HR 0.27) [92].

These findings provide compelling real-world evidence supporting the survival benefits of bezafibrate in combination therapy. Anyway, despite their therapeutic potential, fibrates’ administration has been associated with relevant and non-negligible adverse effects, including rhabdomyolysis, hepatotoxicity, and long-term renal dysfunction [93].

Accordingly, the American Association for the Study of Liver Diseases (AASLD) guidelines recommend avoiding fibrates in patients with advanced decompensated liver disease (Child-Pugh B or C) [94].

Considering these lights and shadows, fibrates have not received formal regulatory approval for PBC, and their use remains off-label in most countries [20,30,94]. Concerns persist regarding long-term safety, particularly in patients with advanced liver disease or renal impairment. Moreover, the heterogeneity of study designs and endpoints across trials complicates direct comparison with approved therapies [20,30,94].

Therefore, although in clinical practice, fibrates have been proposed as an alternative therapeutic strategy in patients with suboptimal response to UDCA, their use should be individualized and closely monitored, ideally within the framework of multidisciplinary hepatology care. Anyway, also in this setting, the indications for fibrates as second-line therapy have been profoundly revised and reframed, in light of the progressive and recent affirmation of novel PPAR ligands [48,84].

##### Next-Generation PPAR Ligands: Status of the Art

Elafibranor is a dual PPAR-α/δ agonist, and thus not associated with the cardiovascular adverse effects typically observed with PPAR-γ ligands. This molecule has been previously investigated in patients with NASH [95] and in murine models of Non-alcoholic fatty liver disease (NAFLD)/Non-alcoholic steatohepatitis (NASH) with liver fibrosis [96]. Interestingly, in rodents, the preclinical molecule (GFT-505) demonstrated protective effects against steatosis, inflammation, and fibrosis through inhibition of pro-inflammatory cytokine [Tumor Necrosis Factor alpha (TNF-α) and Interleukin (IL)-1β] and profibrotic gene expression (tissue inhibitor of metalloproteinases 2, transforming growth factor-β, collagen type Iα1, and collagen type IIα2) [96].

Due to these preclinical encouraging effects and the observed improvements in markers of liver dysfunction, elafibranor was subsequently proposed for evaluation in PBC and tested at moderate-to-high doses (80–120 mg/day) to achieve therapeutic benefit.

In 2021, a randomized placebo-controlled trial by Schattenberg et al. evaluated the efficacy and safety of elafibranor in patients with PBC and inadequate response to UDCA [97]. The trial included 45 adults with ALP levels ≥ 1.67× the ULN, randomized to elafibranor 80 mg, 120 mg, or placebo for 12 weeks. Treatment with elafibranor led to a significant reduction in ALP compared with placebo (−48.3% and −40.6% vs. +3.2%), and higher rates of composite endpoint achievement (ALP ≤ 1.67 × ULN, >15% reduction, and normal bilirubin) in 67–79% of treated patients versus 6.7% with placebo. Moreover, elafibranor improved additional disease activity biomarkers and pruritus symptoms without significant adverse events [97].

These preliminary findings provided the rationale for the phase III ELATIVE trial (NCT04526665), in which 161 PBC patients were randomized (2:1) to receive elafibranor 80 mg/day or placebo, in combination with UDCA if tolerated. In the treatment arm, biochemical response (defined as ALP < 1.67 × ULN with ≥15% reduction and bilirubin ≤ ULN) was achieved in ~51% compared to 4% in the placebo group [98]. Furthermore, normalization of ALP occurred in 15% of elafibranor-treated patients versus none in the control group. Also, the safety profile was favorable: adverse events were comparable across groups, with the most common (≥10%) including abdominal pain, diarrhea, nausea, and vomiting. Based on these results, the FDA granted accelerated approval of elafibranor (Iqirvo^®^, Genfit, Loos, France) as second-line therapy in PBC patients intolerant to UDCA (monotherapy: elafibranor) or presenting with an inadequate response to UDCA (add-on therapy: UDCA plus elafibranor) [99].

Seladelpar is a selective PPAR-δ agonist with anti-inflammatory and choleretic properties, the latter linked to downregulation of bile acid synthesis and modulation of their transport and metabolism. It was first tested in a phase II study including 70 PBC patients with inadequate response to UDCA, randomized to seladelpar 50 mg/day, 200 mg/day, or placebo [100]. Although all patients who completed 12 weeks of treatment achieved normalization of ALP, the study was terminated prematurely due to grade 3 but asymptomatic and reversible aminotransferase elevations in three patients. In response to safety concerns, subsequent studies evaluated lower doses. Firstly, Bowlus et al. conducted a 1-year, open-label, uncontrolled, dose-finding study in 119 patients receiving daily seladelpar at 2 mg, 5 mg, or 10 mg, with dose escalation to 10 mg after 12 weeks depending on biochemical response. After 12 months, no patients remained on 2 mg/day [101]. The composite endpoint (ALP < 1.67 × ULN, ALP reduction ≥ 15%, and normal bilirubin) was reached in 53% of the 5/10 mg arm and 69% of the 10 mg arm. Four patients discontinued treatment due to adverse events, of which two were seladelpar-related (grade 2 aminotransferase elevation and grade 1 heartburn). Notably, no patients reported pruritus. Overall, seladelpar was safe and well-tolerated, and 98% of patients enrolled in the long-term extension study [101].

Subsequently, the phase III ENHANCE trial (NCT03602560) randomized UDCA-inadequate responders to seladelpar 5 mg, 10 mg, or placebo, with a composite endpoint at 12 months (ALP < 1.67 × ULN, ≥15% reduction, and bilirubin ≤ ULN). After 12 months, seladelpar achieved significantly higher biochemical response rates compared with placebo, along with higher ALP normalization rates (25% vs. 0%) [102]. In this study, seladelpar also reduced pruritus in patients with moderate-to-severe symptoms (mean Numerical Rating Scale—NRS-difference −1.5) [102]. Moreover, an open-label longitudinal study confirmed that the biochemical and symptomatic benefits of seladelpar were sustained over two years, with a favorable safety profile and no emergent concerns [103]. In line with this, further reviews and clinical data suggested that seladelpar improves cholestasis, inflammation, and symptoms such as pruritus, although robust evidence of impact on hard clinical outcomes is still lacking [104,105].

More recently, the randomized, double-blind, placebo-controlled phase III RESPONSE trial (NCT04620733), evaluating the efficacy and safety of the molecule in PBC patients who exhibited an inadequate UDCA biochemical response, seladelpar (10 mg once daily) demonstrated superior efficacy compared to placebo, with 62% of treated patients achieving the composite biochemical response endpoint, including normalization of ALP and total bilirubin [106]. Seladelpar also led to significant improvements in pruritus severity and QoL measures, with a favorable safety profile comparable to placebo [106]. Considering these findings, the FDA granted accelerated approval of seladelpar (Lyvdelzi^®^, Gilead Sciences, Foster City, CA, USA) in PBC patients intolerant to UDCA (monotherapy: seladelpar) or presenting with an inadequate response to UDCA (add-on therapy: UDCA plus seladelpar) [107].

Collectively, elafibranor and seladelpar emerged as advanced PPAR agonists for PBC, with robust biochemical efficacy, symptomatic improvement, and sustained tolerability, though their effect on long-term outcomes and optimal role in advanced disease requires further investigations. In this sense, although both elafibranor and seladelpar have demonstrated robust biochemical efficacy and favorable tolerability profiles, important distinctions emerge when considering their broader clinical impact and the uncertainty surrounding long-term outcomes. Elafibranor, as a dual PPAR-α/δ agonist, has shown additional benefits on pruritus and metabolic parameters, suggesting potential systemic advantages beyond cholestasis control [98]. However, long-term data remain limited, and whether these improvements translate into reduced progression to cirrhosis or improved transplant-free survival is still under investigation in emerging clinical trials, including the ongoing “ELFIDENCE” (NCT06016842—“A Long-Term Study of Elafibranor in Adult Participants with Primary Biliary Cholangitis”) [108]. On the other hand, Seladelpar, as a selective PPAR-δ agonist, has consistently demonstrated sustained biochemical response and significant improvement in pruritus and quality-of-life measures, with reassuring long-term safety data from interim analysis presented in extension studies [109,110]. Nevertheless, despite these encouraging findings, neither agent has yet provided definitive evidence of altering hard clinical endpoints such as hepatic decompensation, need for transplantation, or mortality. Future head-to-head studies and long-term randomized controlled trials are required to clarify whether these agents can move beyond biochemical surrogates to deliver tangible improvements in survival and advanced disease outcomes.

### 2.3. Future Therapies in Primary Biliary Cholangitis: A Dynamic and Evolving Drug Pipeline

In recent decades, the therapeutic landscape for PBC has evolved substantially, with several promising agents now emerging on the horizon [77]. In this sense, in parallel with the recent approval of novel PPAR agonists (elafibranor and seladepar), the research efforts have also been focused on the development of further similar molecules in managing PBC [77,84]. Among these, saroglitazar represents one of the most promising drugs.

Saroglitazar is a dual PPAR agonist, predominantly activating PPAR-α with partial PPAR-γ activity. Relevantly, this drug is currently approved outside the United States for hypertriglyceridemia in diabetic patients uncontrolled on statins, as well as for diabetic dyslipidemia and NASH [111].

Anyway, given its mechanism of action, saroglitazar is also under investigation in the PBC setting [77,84]. In a phase II double-blind, randomized, proof-of-concept study (enrolling 37 PBC patients), saroglitazar 2 mg and 4 mg daily for 16 weeks produced significant reductions in ALP [respectively of −155.8 U/L with 2 mg and −163.3 U/L with 4 mg], compared with −21.1 U/L in the placebo group [112]. In this research, the relative ALP reduction was ~49% with 4 mg and 51% with 2 mg, versus ~3% with the placebo. Relevantly, these effects were rapid, evident by week 4, and sustained through week 16 [112].

Safety concerns were noted: 96 treatment-emergent adverse events were reported, with drug-related events occurring in 53.8% of the 4 mg arm, 28.6% of the 2 mg arm, and 30% of the placebo. Four patients permanently discontinued therapy due to asymptomatic aminotransferase elevations (≥5 × ULN in two patients in the 4 mg arm). In three cases, liver enzymes normalized spontaneously within three months of discontinuation, while one patient required immunosuppressive therapy for PBC-AIH overlap [112].

In addition, a phase III open-label multicenter study assessed saroglitazar 4 mg/day plus UDCA for 16 weeks in Mexican PBC patients with inadequate UDCA response, showing a significant mean ALP reduction (−94 ± 53 U/L; ~ −48 ± 23% from baseline) [113].

More recently, pharmacokinetic and pharmacodynamic data analysis of PBC patients with mild/moderate hepatic impairment or severe renal impairment demonstrated that saroglitazar 1–2 mg reduced ALP by 17–40% after 4 weeks, particularly in patients with high baseline values, including those with compensated cirrhosis, without evidence of drug accumulation [114].

In summary, although still in early clinical stages, saroglitazar shows robust biochemical reductions in ALP, with an acceptable safety profile at moderate doses. Larger trials with hard clinical endpoints (disease progression, transplant-free survival) are needed to confirm efficacy and define the optimal dosing strategy that maximizes therapeutic benefit while minimizing hepatic risk.

In addition to agents targeting biochemical response and disease progression, a novel therapeutic pipeline is emerging and specifically addresses patient-reported symptoms, such as cholestatic pruritus and fatigue, which represent major unmet clinical needs in PBC [77]. Several studies have shown that patients with PBC experience the poorest QoL indicators among autoimmune liver diseases, with higher levels of fatigue, sleep disturbance, and depression compared with other populations [115]. Disease-specific instruments such as the PBC-40, alongside other validated QoL questionnaires, consistently demonstrate that chronic pruritus exerts a devastating influence on daily functioning and psychosocial well-being [116,117]. Importantly, patient self-reported experiences highlight that, despite improved survival with current therapies, pruritus remains one of the most debilitating manifestations of PBC, affecting up to 80% of individuals during the disease course [116,117]. Notably, the severity of pruritus does not correlate with histological stage or biochemical indices, emerging as a symptom often disproportionate to the underlying hepatic injury [118,119]. Furthermore, UDCA, the cornerstone of PBC therapy, has shown no significant impact on the management of pruritus, either beneficial or detrimental [120]. Given that antihistamines—despite their widespread clinical use—prove ineffective in most cases, current international guidelines advocate for a structured, stepwise approach to their symptomatic management [20,30,94].

Bile acid sequestrants, such as cholestyramine and colesevelam, remain the recommended first-line therapy for cholestatic pruritus in PBC, with colesevelam generally better tolerated, although both are limited by gastrointestinal adverse effects [121].

In patients with inadequate response, rifampicin is considered the most effective second-line option, but its use requires close biochemical monitoring given the risk of hepatotoxicity and hemolytic anemia [122]. Third-line approaches include opioid antagonists such as naltrexone, which has demonstrated superiority over placebo in a meta-analysis [123]; however, the potential for withdrawal-like reactions necessitates a cautious “low and slow” titration strategy [120]. Sertraline has also emerged as a potential adjunct, with several studies indicating modest antipruritic efficacy independent of its antidepressant properties, likely mediated through serotonergic modulation of nociception [124,125,126].

Despite this therapeutic armamentarium, the limited efficacy and suboptimal tolerability of current strategies underscore the urgent need for novel interventions [120]. Among emerging options, fibrates have gained considerable interest.

A recent systematic meta-analysis demonstrated a significant reduction in pruritus among PBC patients treated with fibrates, although data remain largely derived from small-scale studies [119,127]. Bezafibrate, in particular, has shown symptomatic improvements in case series and pilot trials, suggesting a potential modulatory effect on pruritogenic pathways [90].

More compelling evidence is now available for next-generation PPAR agonists. Seladelpar has not only improved biochemical indices of cholestasis but also achieved a significant reduction in pruritus, an effect associated with decreased circulating levels of interleukin-31, a key mediator of itch signaling [128].

In parallel, ileal bile acid transporter (IBAT) inhibitors have introduced a novel mechanistic paradigm. Linerixibat, an oral IBAT inhibitor, demonstrated significant efficacy in the global phase III GLISTEN trial (NCT04950127): over 24 weeks, the mean worst-itch numerical rating scale (WI-NRS) score improved, with rapid onset by week 2 and sustained relief, including reductions in itch-related sleep interference [129]. Moreover, Linerixibat demonstrated clinically meaningful improvements in itch severity and sleep quality in the phase III GLIMMER trial (NCT04603937), highlighting modulation of the enterohepatic circulation of bile acids as a promising target [130].

Additionally, the ongoing phase 1b/2 trial is evaluating the efficacy of golexanolone, a neurosteroid modulator acting on Gamma-Aminobutyric Acid-A (GABA_A_) receptors, in targeting fatigue, a frequent and impactful symptom in PBC. Though primarily investigated in hypersomnia and hepatic encephalopathy, its mechanism may alleviate fatigue, highlighting a new and promising therapeutic frontier [131].

Collectively, these advances mark a paradigm shift in the management of pruritus in PBC—from purely symptomatic control toward mechanism-based therapies—offering the prospect of more effective and better-tolerated interventions for this highly debilitating symptom. Future challenges include long-term validation of clinical benefit (beyond surrogate endpoints), integration into treatment algorithms, and alignment with regulatory pathways, particularly given the epidemiology of PBC and the evolving standards for accelerated approvals.

In this sense, the experience with OCA has provided critical insights for future drug introduction in the PBC setting, including the necessity for more robust trial design, careful patient selection, and validated surrogate endpoints to ensure the achievement of both regulatory approval and long-term clinical benefit [79].

Table 1, by reporting the relative targets, mechanisms of action, and key trials, summarizes the state of the art of the molecules approved and/or under investigation in the treatment landscape of PBC (Table 1).

## 3. Predicting Disease Course in Primary Biliary Cholangitis in the Era of Noninvasive Tools

### 3.1. Role of Demographic and Clinical Profile

Age and sex have been largely demonstrated to influence both response to treatment and long-term outcome of patients with PBC, thus enabling the inclusion of these variables into various risk stratification and prognostic models for PBC, as detailed below [132,133,134]. In particular, although PBC is predominantly a female disease, male patients often present with a specific clinical profile, characterized by later diagnosis, more advanced disease stage at presentation, and a poorer biochemical response to UDCA therapy compared to females, with reported response rates of 72% for males versus 80% for females [132,133,134]. Furthermore, male sex emerged to be associated with a higher risk of developing HCC and increased rates of liver-related mortality [135,136,137]. Concerning this, a large population-based study from Italy and Denmark showed that males had worse survival than females [HR for all-cause mortality 2.36 in the Italian and 3.04 in the Danish population], although a multivariable analysis with inclusion of liver biochemistry or disease severity was not performed [138].

However, the role of male sex as an independent prognostic factor remains a subject of debate, with emerging observations suggesting that the differences in biochemical response and outcomes according to sex may be attributed to males presenting at a more advanced disease stage [132,139]. Consistently, in the multivariate analysis performed by Trivedi et al., male sex was not found to be a significant predictor of liver transplant or death at diagnosis [140].

On the other hand, the impact of age is multifaceted. It is well established that younger age at diagnosis is associated with a more symptomatic presentation and predicts a reduced likelihood of achieving a biochemical response to UDCA, contrariwise to older patients [132,133,134].

Consistently, the UK-PBC study group showed an inverse correlation between age at diagnosis and achievement of biochemical response at 1 year of UDCA therapy in a large cohort of PBC patients, with the lowest response rate in individuals younger than 30 years (<30%), while patients diagnosed over the age of 70 achieved biochemical response rates up to 90% [132].

Potential mechanisms for age-related effects include hormonal effects, such as elevated oestrogen levels in younger women leading to treatment resistance, and aspects of immune senescence [141].

In parallel, despite a potential advantage to UDCA response, older age itself is also an independent risk factor for adverse outcomes, including liver transplantation or death [140]. In support of this, Trivedi et al. demonstrated that each additional year of age at diagnosis was associated with a significantly higher risk (HR: 1.06) of these outcomes [140].

Nowadays, reflecting earlier detection of PBC, most patients are typically diagnosed when asymptomatic. When symptoms are present, the most reported include fatigue, itching, and sicca complex, significantly burdening patients’ QoL [142,143,144,145]. There is conflicting evidence regarding the direct prognostic effect of symptoms. A retrospective cohort study of 216 patients showed symptomatic presentation may be associated with younger age, a poorer response to UDCA therapy (63% vs. 81%, respectively), and an increased risk of cirrhosis and its complications (31% vs. 13%, respectively) [146]. Furthermore, a longitudinal prospective study revealed that patients experiencing fatigue had significantly worse survival outcomes, independent of UDCA response and disease severity [147].

These findings suggest that the presence or development of symptoms may act as a surrogate marker for a more advanced histological stage or a more aggressive disease subset. However, it is crucial to differentiate the prognostic significance of the presence of symptoms from the severity. European Association for the Study of the Liver (EASL) guidelines explicitly state that the severity of PBC-related symptoms does not necessarily correlate with the stage of the underlying liver disease [20].

Specifically, while pruritus has been reported to improve as liver disease worsens, fatigue is not related to the severity of liver disease, except in the case of end-stage liver disease (ESLD), where it is consistently present [20]. Therefore, while the initial symptomatic status or the development of symptoms during the disease course can be a relevant prognostic indicator, reflecting a more progressive disease trajectory, the severity of single symptoms, like pruritus and fatigue, should not be used as a primary or reliable determinant of the liver disease progression or overall long-term prognosis.

A notable exception, denoting a clinically relevant subset, is the presence of severe itching, along with cholestatic jaundice, which should raise suspicion for a premature ductopenic variant (detailed below), affecting approximately 5–10% of PBC patients and significantly impacting prognosis [137,148].

Conclusively, a comprehensive initial assessment, evaluating factors such as male sex and younger age at onset (<45 years), is essential for accurate risk stratification, as these are recognized baseline predictors of poorer outcomes. The potential additional prognostic value of symptoms within current risk stratification models remains uncertain and warrants further evaluation through prospective studies.

### 3.2. Role of Routine Hepatic Tests

Among biochemical parameters, ALP and bilirubin are considered the strongest independent predictors of long-term prognosis, and the most robustly validated markers of disease activity (ALP and bilirubin) and disease stage (bilirubin) in PBC, with a predicting power that remains correlated with clinical outcomes over time [20,43,149,150]. ALP is the most widely recognized marker of therapeutic response, also correlating with the two hallmarks of biliary injury, ductular reaction and biliary metaplasia, which are determinants of responsiveness to choleretic treatment [20,43,149,150]. Both reduction and normalization of ALP are associated with improved prognosis and reduced risk of liver-related complications [20,43,149,150]. An elevated basal ALP level has been associated with a lower likelihood of achieving a deep response to UDCA, as well as consistently, ALP serum level > 1.5–1.67 × ULN, despite the therapy, is strongly associated with a higher risk of progression [20,43,149,150].

Consistently, in a large international cohort of 1047 patients after at least 12 months of UDCA, Corpechot et al. showed that normalization of ALP was obtained in a subgroup of individuals with significantly improved long-term outcomes compared with patients who only met the broader definition of biochemical response [151]. Specifically, patients with normal ALP (66%) experienced an estimated gain of 7.6 months in 10-year complication-free survival compared with those with residual ALP elevation [151].

In addition, stratifying by liver stiffness measurement (LSM) and age, a prognostic advantage of ALP normalization emerged in patients with higher LSM (≥10 kPa) and younger age (≤62 years), where the survival benefit extended to more than 50 months over 10 years [151]. These findings highlight the concept of a “deep biochemical response,” underscoring that normalization of ALP, rather than partial reduction, should be considered as an optimal therapeutic target in PBC management [151].

Serum bilirubin is an additional critical index of therapeutic response, a prognostic indicator, and a surrogate marker of ductopenia, with even minimal increases being strongly predictive of adverse outcomes [150,152].

More than forty years ago, Shapiro et al. first recognized the association between serum bilirubin levels and survival, with higher levels predicting shorter survival times, identifying three main thresholds associated with progressively lower survival: >2 mg/dL, >6 mg/dL, and >10 mg/dL, respectively, in two subsequent measurements within 6 months [153].

Moreover, their results, subsequently implemented by longitudinal large-scale analyses, have identified two distinct phases in bilirubin progression: an initial stable phase lasting several years, followed by a rapid “acceleration” phase preceding clinical endpoints such as liver transplant or death, identifying a threshold at approximately 1.6 times the ULN, beyond which the risk of clinical events increases sharply, typically within less than two years [153].

These findings suggest that bilirubin may represent a “late” biomarker, primarily reflecting end-stage disease progression, contrariwise to ALP, which is considered an early marker of the disease.

Furthermore, in a large multicentric cohort of PBC patients, Murillo-Perez et al. demonstrated that achieving low thresholds of serum bilirubin (≤0.6 × ULN), as well as normalization of ALP (ALP ≤ 1 × ULN) at one year of therapy, is associated with significantly improved transplant-free survival [152]. Specifically, the 10-year survival rate was 91.3% for bilirubin ≤ 0.6 × ULN versus 79.2% for levels above this threshold, while normalization of ALP corresponded to a 10-year survival of 93.2%, compared to 86.1% in patients with ALP between 1.0 and 1.67 × ULN [152]. Consistently, in a meta-analysis of 4845 patients, Lammers et al. showed that both ALP (>2.0 ULN) and bilirubin (>1.0 × ULN) are independent predictors of liver transplant and death [150].

Further biochemical parameters, such as aspartate aminotransferase (AST), alanine aminotransferase (ALT), and GGT, have been included in composite scores, considering their specificity as individual markers is reduced, as well as low albumin levels, prolongation of prothrombin (together with raised bilirubin values) are features of advanced cirrhosis without a significant role as independent markers [20,30,154].

In detail, despite an increase in GGT may precede that of ALP in initial stages of PBC, baseline GGT levels themselves do not appear to be associated with disease progression [155]. However, a reduction of approximately 70% or normalization within six months of initiating UDCA therapy has been linked to a more favorable prognosis [156]. In a prospective multicenter cohort of 297 patients, Ter Borg et al. demonstrated that stratifying patients according to the combination of bilirubin and albumin allows the identification at baseline of UDCA-treated PBC patients that are at low (both normal bilirubin and albumin), median (abnormal bilirubin or albumin), and high risk (abnormal bilirubin and albumin) of future clinical events [157]. In the low-risk group, liver transplant-free survival was comparable to that of age- and sex-matched general population, suggesting a potential disease-modifying effect of UDCA in the early-stage of PBC [157]. Conversely, patients with median and high risk exhibited significantly reduced survival, underscoring the prognostic value of these biochemical markers [157]. Multivariate analysis confirmed bilirubin as the strongest predictor of outcome, followed by albumin and platelet count [157]. Moreover, serum albumin, among its heterogeneous applications in the risk stratification of PBC patients, recently emerged also as a robust and accessible biomarker of clinically significant portal hypertension (CSPH), particularly in settings where transient elastography is unavailable or unreliable due to cholestatic interference (see the dedicated paragraph for details) [158].

### 3.3. Role of Serological Markers: Defining the Impact of PBC-Associated Autoantibodies

Although AMA autoantibodies are considered the serological hallmark for diagnosis of PBC, neither their presence nor their titer has been demonstrated to be associated with the prognosis [137,148,159]. Therefore, once a definitive diagnosis has been established, repeated measurement is not advised, as UDCA treatment may lower AMA levels and potentially introduce unwarranted interpretative ambiguity [30].

However, several studies suggested that AMA-negative PBC patients have poorer outcomes compared to AMA-positive cases, with a lower survival rate as well as higher liver-related complications, including liver transplant and death [137,148,160].

ANA autoantibodies, found in about 30% of PBC patients and up to 50% in AMA-negative patients, hold significant prognostic value [28,137]. Specifically, anti-gp210 positivity correlates with higher baseline transaminases and bilirubin levels, increased LSM values, a reduced treatment response to UDCA, a six-fold increased risk of progression to liver transplant or death due to hepatic failure, and a lower 5-year transplant-free survival rate [161,162,163,164].

Consistently with previous research, in a cohort of almost 500 patients, Haldar et al. observed that individuals positive for anti-gp210 antibodies exhibited a significantly lower response rate to UDCA therapy compared to those who were negative (39.3% vs. 16.7%), sustaining the potential utility of anti-gp210 as a predictive marker for suboptimal UDCA response, with a sensitivity of 26.8% and specificity of 89.8% [162].

The treatment response remains unsatisfactory for anti-gp210-positive patients also in the case of second-line therapeutic strategies [159,165]. Therefore, the inclusion of anti-gp210 antibodies into the UK-PBC and GLOBE scoring systems could potentially enhance their risk stratification capabilities [166].

The prognostic relevance of anti-gp210 antibody titers was further investigated by Nakamura et al., revealing that patients whose levels declined following UDCA therapy exhibited milder disease and more favorable outcomes compared to those with stable or increasing titers [167]. However, the variability of anti-gp210 concentration over time remains controversial, as further studies have observed no significant changes during long-term follow-up [164,168]. Concerning this, a comparative analysis of detection methods revealed that fluctuations in antibody titers were detectable when the carboxy-terminal tail of gp210 was used as the assay substrate, whereas studies reporting stable levels often lacked methodological details regarding antigenic targets [160]. This discrepancy underscores the importance of assay standardization in interpreting serological markers.

The prognostic role of sp100 antibodies remains a subject of ongoing discussion: despite some studies previously demonstrating a correlation between their presence and disease severity/treatment response, their role as an independent prognostic marker has not been established to date [58,169,170,171].

The presence of anti-centromere antibodies (ACAs) is observed in approximately 9–30% of PBC patients and has been associated with potential prognostic implications, with particular reference to a recognized association with portal hypertension (PH) progression and severe bile duct injury [167,172]. Ding et al. recently demonstrated that the combined serological profile, defined as the simultaneous presence of anti-gp210 and ACA, is an independent prognostic marker of poor prognosis and may enhance risk stratification when integrated with established scoring systems, such as the GLOBE score [173].

Beyond traditional autoantibodies, novel emerging markers offer further prognostic insights. Anti-hexokinase 1 (HK1) antibodies revealed a specific association with a higher likelihood of LREs and lower transplant-free survival, while anti-Kelch-like 12 (KLHL12) antibodies showed a link to advanced liver fibrosis and elevated bilirubin levels [174]. However, these novel markers require validation in larger and heterogeneous cohorts.

### 3.4. Role of Histological Features

Although the role of liver biopsy has been drastically reduced over the years and is now principally limited to seronegative forms, disease variants [e.g., PBC-Autoimmune hepatitis (AIH) variant], or resistance to UDCA therapy, histological evaluation still offers critical insights into disease severity, progression, and prognostic implications [20].

Among the most relevant histological markers, the florid duct lesion, although considered specific to PBC, is identified in only a minority of biopsy specimens (~10%) and tends to appear in early disease stages [20,148]. On the other hand, ductopenia, defined as the loss of interlobular bile ducts, is a well-established predictor of disease progression [58,156,175]. Consistently, its presence correlates with worse cholestasis, poor response to therapy, and a more rapid transition to ESLD [58,156,175]. The so-called “premature ductopenic variant” of PBC, often presenting with severe pruritus and jaundice in non-cirrhotic patients, has been linked to fast clinical deterioration and early need for liver transplant [58,156,175].

Particular mention should be given to interface hepatitis, characterized by lymphocytic infiltration at the portal–parenchymal interface, which may indicate the presence of PBC-AIH variant, especially in patients with elevated ALT and IgG levels [176]. Emerging evidence has shown that moderate-to-severe interface activity is associated with increased risk of cirrhosis and liver-related mortality, highlighting the role of interface hepatitis as an independent prognostic factor in PBC [176,177,178].

Ductular reaction, representing a proliferative response to biliary injury, has also gained attention for its prognostic implications [148]. Recent data suggest a strong correlation between the extent of ductular reaction, ALP levels, and UDCA responsiveness, indicating that histological evidence of biliary regeneration may reflect ongoing injury and influence treatment outcomes [179].

Consistent with the natural history of chronic liver diseases (CLDs) of various etiology, histological progression reflects cumulative hepatic injury and impaired regenerative capacity.

Accordingly, advanced histological stages of liver disease, specifically extensive fibrosis or cirrhosis, independently predict poor prognosis, representing strong determinants of survival expectation and negative outcomes [137,148].

Beyond traditional histological staging system, such as Ludwig and Scheuer, affected by limited reproducibility and prognostic accuracy, the Nakanuma system proposed by Harada et al., incorporating fibrosis, bile duct loss, and deposition of orcein-positive granules, has demonstrated a superior prognostic ability and correlation with clinical outcomes/treatment failure, thanks to the introduction of the concept of PBC grading, evaluating cholangitis activity and hepatitis activity [180,181,182]. However, its utility is constrained by inter-observer variability in staging assessment and limited concordance in evaluating necro-inflammatory activity.

An alternative histological scoring system proposed by Wendum et al., which independently evaluates fibrosis, ductopenia, and interface hepatitis, has demonstrated stronger correlation with biochemical parameters and improved reproducibility among observers [183]. Nonetheless, its prognostic applicability remains uncertain due to the limited duration of follow-up in validation studies [183].

Taking into account all the above-mentioned considerations, despite histological assessment continues to play a central role in refining prognostic stratification and guiding individualized therapeutic strategies, the limitations related to the intrinsic invasiveness of the procedure and sampling variability, and the widespread adoption of prognostic non-invasive tools (NITs) have drastically restricted the applicability of liver biopsy in routine clinical practice, reserving this strategy to atypical PBC-settings and clinical research purposes.

### 3.5. Role of Noninvasive Tools

In recent years, scientific efforts have focused on the development and implementation of NITs to define liver fibrosis and disease progression status, intending to improve the long-term monitoring of CLD severity and identify high-risk patients who may benefit from alternative or intensified therapeutic approaches by employing simple, reproducible, and reliable low-cost evaluations.

#### 3.5.1. Role of Biomarkers

Serum hyaluronate was the first marker shown to closely correlate with histopathological liver changes in PBC, significantly increasing across different disease stages [184]. Mayo et al. have further highlighted the potential role of serum hyaluronate as a marker of disease progression status by elaborating the Enhanced Liver Fibrosis (ELF) score, also involving procollagen III peptide and tissue inhibitor of metalloproteinase 1 [185]. Recent data confirmed the good performance (AUROC: 0.86) of the ELF score in predicting adverse outcomes (liver-related complications/mortality) in the PBC context, and the highest correlation, compared to other serum indices, with liver fibrosis evaluated according to either the Scheuer or Nakanuma classification [186].

However, the longitudinal interpretation of ELF score changes over time remains challenging, as its correlation with disease dynamics is not yet fully elucidated, and it appears to be less effective in patients with early disease [185]. Moreover, its limited implementation in routine clinical practice can be attributed not only to its relatively high cost but also to the potential variability due to demographic factors such as gender, age, and ethnicity, which may affect test performance and interpretation.

The AST-to-platelet ratio index (APRI) score has emerged as an effective, easy-to-use, low-cost surrogate marker of liver fibrosis and portal hypertension, which are critical determinants of patient outcomes [43,140,187].

In a large multicenter cohort of PBC patients, an APRI > 0.54 after one year of UDCA treatment accurately predicted the risk of major liver-related complications within 10 and 15 years beyond biochemical response to UDCA assessed by GLOBE score, enhancing its prognostic performance [175]. However, APRI, similarly to other NITs, such as FIB-4, the AST/ALT ratio (AAR), and red blood cell distribution width to platelet ratio (RPR), has shown a suboptimal diagnostic performance (AUROC < 0.80) in predicting histological stage in the specific setting of PBC [188,189,190,191,192,193,194].

In fifty-eight treatment-naïve PBC patients, Wang et al. demonstrated that the platelet count to spleen thickness ratio (PC/ST ratio) has a good diagnostic performance in predicting advanced fibrosis stage, although further validation is warranted [195].

In PBC patients, PH can develop even in the early stages of disease, either in association with or independently of nodular regenerative hyperplasia, and the presence of a pre-sinusoidal component may lead to an underestimation of the actual portal pressure as measured by the hepatic venous pressure gradient (HVPG) [196]. In this regard, in a multicenter cohort of 293 patients with compensated advanced chronic liver disease (cACLD), Calvaruso et al. showed that the RESIST criteria, based on platelet count and serum albumin levels, demonstrated a superior diagnostic performance compared to elastography-based algorithms, such as Baveno VI and Expanded Baveno VI, in the non-invasive assessment of CSPH in the specific setting of PBC [158].

Specifically, patients with serum albumin levels ≥ 3.6 g/dL and platelet count ≥ 120 × 10^9^/L were classified as low risk for high-risk varices (HRVs), with a false-negative rate of only 2.5%, significantly lower than that observed with elastography-based criteria [158]. Moreover, the study highlighted that elevated ALP values (≥1.5 × ULN) negatively impacted the performance of all NITs, while RESIST criteria maintained the predictive accuracy even in this subgroup [158].

Finally, in this scenario, Wisteria floribunda agglutinin-positive Mac-2 binding protein (WFA + −M2BP) represents another promising simple, non-invasive serological marker for liver fibrosis and prognosis in PBC, though further validation is required [197].

#### 3.5.2. Role of Elastography-Based Methods

The increasing focus on non-invasive strategies to assess disease severity in PBC patients, overcoming liver biopsy limitations, at baseline and throughout follow-up, has led to the widespread adoption of imaging-based tools for assessing liver fibrosis and disease progression. Among these, LSM by transient elastography (TE) has emerged as a cornerstone opportunity, offering reproducible and prognostically meaningful data.

In a large international multicenter retrospective study involving 3985 PBC patients, LSM was independently associated with poor clinical outcomes, with adjusted hazard ratios (aHRs) per additional kilopascal (kPa) ranging from 1.040 to 1.042 [198]. Notably, LSM values between 5 and 30 kPa demonstrated a monotonic increase in the risk of adverse events, underscoring its utility in longitudinal risk stratification [198]. Based on these findings, thresholds of 8 kPa and 15 kPa have been proposed to categorize patients into low-, moderate-, and high-risk groups, with approximately 40% of individuals falling into the latter two categories [198]. Moreover, the predictive value of LSM remains stable over time and complements existing biochemical response criteria and prognostic models [198]. Importantly, emerging findings have demonstrated that LSM by TE represents the best surrogate marker for ruling in severe fibrosis. Concerning this, the cut-off of 9 kPa has been suggested to differentiate patients with significant fibrosis, while values around 10.7 kPa may indicate advanced fibrosis [198].

A smaller study involving 44 PBC patients confirmed the high diagnostic accuracy of LSM by TE, reporting AUROCs of 0.91 and 0.97 for advanced fibrosis and cirrhosis, respectively [199].

To date, the EASL guidelines recommend a threshold of 10 kPa to rule in advanced fibrosis, though optimal cut-offs may vary depending on the population and technique used [200].

Beyond static measurements, dynamic changes in LSM over time offer additional prognostic insight. Consistently, LSM monitoring during therapy administration is increasingly recognized as a valid predictor of long-term clinical outcomes, encompassing LREs, LT, or death, even independently of baseline evaluation [201]. An annual increase of 2.1 kPa in LSM has been associated with an 8.4-fold higher risk of adverse outcomes [201]. Although the optimal interval between assessments remains undefined, current recommendations suggest repeating LSM every two years in early-stage disease and annually in advanced cases [200].

LSM also plays a pivotal role in the non-invasive evaluation of PH, further enhanced by the combination with spleen stiffness measurement (SSM), whose combination significantly improves risk stratification for liver decompensation and potentially reduces the need for unnecessary diagnostic endoscopies [196]. Consistently, SSM has demonstrated a strong predictive value for PH and variceal risk: thresholds below 40–46 kPa can effectively exclude HRVs, and the integration of SSM with LSM and platelet count further refines risk stratification [202]. Conversely, a combination of LSM > 10 kPa and SSM > 40 kPa strongly predicts liver decompensation [202].

However, despite this evidence, LSM is subject to several limitations. LSM elevations may occur due to inflammation, cholestasis, recent food intake, or venous congestion, necessitating a minimum fasting period of three hours before measurement [200]. Additionally, VCTE performance may be compromised in obese individuals (Body Mass Index—BMI—>28 kg/m^2^), although XL probes offer partial mitigation [200].

In contrast, magnetic resonance elastography (MRE) provides deeper tissue penetration and is unaffected by body habitus. Interestingly, MRE has shown superior accuracy in detecting advanced fibrosis (cut-off: 4.60 kPa; AUROC: 0.82) and predicting hepatic decompensation (HR: 2.09). However, its higher cost and limited availability restrict its use to selected cases, such as patients with elevated BMI or those undergoing cross-sectional imaging for other indications. While preliminary data are encouraging, further validation in larger and more diverse cohorts is needed [200,203,204,205].

Conclusively, elastography-based methods, particularly LSM and SSM, are reshaping the landscape of non-invasive assessment in PBC. Their integration into routine clinical practice enhances risk stratification, informs therapeutic decisions, and aligns with the broader goals of precision medicine. Future research should focus on refining cut-off values, validating dynamic monitoring strategies, and expanding the applicability of advanced imaging techniques such as MRE to optimize patient outcomes.

### 3.6. Predicting Treatment Response in Primary Biliary Cholangitis: An Evolving Challenge

Introduced by Dickson et al. in 1989, subsequently modified in 1994, further simplified in 2000, and extensively cross-validated in other cohorts, the Mayo Risk Score (MRS) was developed to predict prognosis in PBC patients, with the primary objective to enhance patient selection and optimize the timing for liver transplantation before the introduction of UDCA as mainstay treatment [206,207,208]. The MRS is based on a combination of five readily available clinical and biochemical variables: patient age, serum albumin, total bilirubin, prothrombin time, peripheral oedema (or the use of antidiuretic therapy), and predicts survival up to 7 years of follow-up; a threshold of 7.8 of the MRS has been identified as optimal for guiding liver transplant decisions [206,207,208].

Even before the notorious MRS, the Yale and the European models were validated as survival prediction models, both including not only clinical and biochemical variables but also histologic parameters [209,210]. However, the invasive nature due to the necessity of a liver biopsy made these tools impractical for routine use [209,210].

Further general models used for ESLD are the albumin–bilirubin score (ALBI), Child–Turcotte–Pugh (CTP) score, and the Model for End-stage Liver Disease (MELD) [211,212,213]. Subsequently, with the widespread availability of AMA testing, leading to earlier diagnosis, and the introduction of UDCA therapy, which substantially improved the disease course, prognostic scores that included surrogate markers of ESLD, as well as the need for liver biopsies, or the use of subjective parameters (e.g., peripheral oedema), became less relevant or even obsolete.

In the post-UDCA era, it appeared crucial to concentrate scientific efforts on more refined and dynamic prognostic tools that could account for treatment response in the modern therapeutic PBC landscape. In this sense, the introduction of UDCA therapy in the late 1990s marked a paradigm shift in the natural history of PBC, since the biochemical response to treatment emerged as the strongest predictor of long-term outcomes. This pivotal evidence empowered the development of several treatment-response criteria, which currently rely exclusively on surrogate biochemical parameters.

#### 3.6.1. Composite Models Predicting Treatment Response in PBC

In the PBC setting, the slow natural history, simultaneously with its initial indolent course, traditionally makes the use of hard clinical endpoints (including hepatic decompensation, liver transplantation, or death) not suitable to assess therapeutic efficacy and to predict long-term prognosis within the time frame of most clinical trials.

Consistently, to overcome this limitation, multiple surrogate biochemical markers have shown a well-established relationship with long-term outcome, as well as disease activity and stage in PBC, providing an easily available way to identify patients who are likely to benefit from additional therapies [44].

The earliest treatment-response criteria introduced were based on the qualitative assessment of the biochemical response to UDCA, enabling the binary dichotomization of patients as responders or non-responders, and the direct prediction of survival outcomes (liver-related mortality and the need for liver transplantation), except for the Toronto criteria, which predict histologic progression [43,214,215]. Specifically, by evaluating certain thresholds for biochemical parameters (ALP, bilirubin, serum albumin, and transaminases) after 6 to 24 months of therapy with a stable, optimized UDCA-dose (13–15 mg/kg/day), several prognostic models were developed and extensively validated over the years.

Currently, the most widely adopted models are the Paris I, Paris II, Barcelona, Rotterdam, and Toronto criteria, currently endorsed for risk stratification in routine clinical practice [43,214,215].

Among these, the Paris I criteria, defining response as ALP < 3 times the ULN, AST < 2 times ULN, and bilirubin < 1 mg/dL (or <17 µmol/L) after 12 months of UDCA, have often been reported as accurate, exhibiting a high rate of transplant-free survival (90% at 10 years), as well as a good performance in discriminating between low- and high-risk patients across numerous large independent cohorts [58,215,216,217,218]. In a retrospective single-center study of 153 PBC patients, evaluating the predictive performance of different biochemical response criteria to UDCA, Paris I demonstrated to have the best AUROC (0.81–0.82) among qualitative indices [218]. Consistently, a recent systematic review and meta-analysis by Gazda et al., UDCA failure, according to Paris-1, was associated with LREs occurrence (HR 4.22) [219].

The Paris II criteria were specifically designed for patients with early stages of PBC, well reflecting the majority of recent cohorts, and define response as ALP and AST ≤ 1.5 times ULN and normal bilirubin levels after 1 year [218].

According to the above-mentioned meta-analysis by Gazda et al., the risk for LREs in patients experiencing UDCA-failure, based on Paris II criteria, was significantly elevated (HR: 4.47) [219].

The Barcelona criteria define response as an ALP decrease greater than 40% from baseline or normalization within 12 months, and, according to this model, UDCA-failure is associated with LREs occurrence (HR 1.95) [58,214,215,216,218].

The Rotterdam criteria are based on the evaluation of albumin and bilirubin values after 12 months of UDCA treatment and primarily predict liver-related outcomes (HR 4.16, for liver transplant or death vs. HR 2.98 for LREs) [58,175,214,215,216,218,219].

The Toronto criteria define response as ALP < 1.67 times ULN after 24 months, aiming to predict histological stage progression [58,175,214,215,216,218,219]. In subgroup analyses of the research by Gazda et al., UDCA failure according to Toronto was associated with LREs occurrence in PBC patients (HR 3.13) [219].

Despite most criteria evaluating biochemical response after 12 months of UDCA, increasing evidence suggests that the evaluation at 6 months might be more effective because it allows an equivalent prediction reliability with a shorter delay in stratifying patients’ treatment response [44]. Concerning this, Leuschner et al. demonstrated that approximately 80% of the decrease in serum ALP, the most widely recognized marker of therapeutic response, during UDCA treatment occurs within the first 6 months, suggesting that most criteria are best applied after at least 6 months of therapy [66].

Recently, Koky et al. showed that the achievement of a biochemical response after 6 months of UDCA therapy is associated with lower rates of disease progression and liver-related mortality [45]. In contrast, non-responders at 6 months showed a markedly increased risk of adverse outcomes, supporting the importance of early stratification and timely initiation of second-line treatments [45]. For instance, an ALP level exceeding 1.9 times ULN at 6 months has been identified as a reliable predictor of insufficient response (defined by POISE criteria) at 1 year and identifies up to 67% of non-responders earlier [152].

However, dichotomous response models inherently oversimplify the complexity of disease progression [43,148,177]. By reducing continuous biochemical variables to binary categories, these frameworks risk discarding valuable prognostic information and falsely suggesting the existence of only two discrete risk levels [43,148,177]. Moreover, they lack temporal resolution, failing to capture the dynamic evolution of risk over time [43,148,177].

To overcome the abovementioned limitations, the UK-PBC Research Group and the Global PBC Study Group, by analyzing large, multicenter PBC cohorts, independently proposed 2 continuous scoring systems (the UK-PBC risk score and the GLOBE score, respectively), offering higher predictive ability and precision at an individual level [177,220].

These models, by incorporating both biochemical measures of treatment response and parameters of disease severity, provide a probability of transplant-free survival or risk of LREs on a continuous scale, allowing for a more nuanced risk stratification [177,220].

Specifically, the GLOBE score takes into account age at baseline, serum albumin, bilirubin, ALP, and platelet count after 12 months of UDCA treatment, and predicts LT-free survival at 3, 5, 10, and 15 years [177].

In parallel, the UK-PBC score includes baseline albumin and platelet count, bilirubin, ALP, and transaminases after 12 months of UDCA treatment, and estimates the risk of LT or liver-related death occurring within 5, 10, or 15 years [220].

Notably, both the GLOBE and UK-PBC risk scores have shown enhanced predictive accuracy for liver-related mortality and LT when compared to the Paris-I criteria, with 15-year c-statistics of 0.82 and 0.90, respectively, in external validation cohorts [166].

In a large United States cohort of UDCA-treated PBC patients, both the GLOBE and UK-PBC risk scores demonstrated excellent prognostic accuracy in predicting treatment response (AUROCs of 0.87 and 0.94, respectively) and outperformed the Paris II criteria, thereby confirming their clinical utility for individualized risk stratification and guiding therapeutic decisions [63]. Recent meta-analytic data have confirmed the strong prognostic performance of the GLOBE and UK-PBC risk scores in predicting liver-related outcomes in PBC [219]. The continuous form of the GLOBE score demonstrated HRs for LREs ranging from 3.03 to 5.05, while its binary form yielded a markedly higher but less stable HR of 58.83, reflecting statistical imprecision [219]. Similarly, the continuous UK-PBC score showed an HR of 3.39, outperforming its binary counterpart (HR: 2.76). Both models exhibited excellent discriminative ability, with AUROC values ranging from 0.83 to 0.94 for GLOBE and 0.79 to 0.95 for UK-PBC across 10- and 15-year time horizons [219].

Despite their strong prognostic performance, the GLOBE and UK-PBC risk scores present several limitations that constrain their clinical applicability. Notably, no universally accepted thresholds have been established to clearly distinguish high-risk from low-risk patients, limiting their use in guiding treatment escalation or de-escalation [148]. Furthermore, their predictive accuracy in patients receiving second-line therapies needs further validation in larger, real-world cohorts [148].

Additionally, both scores cannot identify individuals unlikely to respond to UDCA before treatment initiation, opening the doors to further emerging models for pre-therapy prediction of response [148].

Table 2 summarizes the current validated model to predict therapeutic response (after starting therapy) in PBC (Table 2).

#### 3.6.2. Models for Pre-Treatment Prediction of Response: Role of Multivariate Risk Scores

To address the pre-treatment prediction of response to therapy, the UK-PBC Research Group and Italian PBC Study Group have developed the UDCA Response Score (URS), integrating several pre-treatment clinical and serologic variables to discriminate PBC patients who are unlikely to respond to UDCA monotherapy before treatment initiation (AUROC 0.83) [141]. Specifically, the URS incorporates six pre-treatment variables: ALP, total bilirubin, transaminases, age at diagnosis, time interval between PBC diagnosis and initiation of UDCA therapy, and the delta (∆) change in ALP levels from diagnosis to treatment onset. Elevated ALP at baseline, a subsequent rise in ALP before starting UDCA, increased bilirubin, younger age, and a prolonged delay in initiating treatment were all associated with a reduced likelihood of biochemical response.

In contrast, higher transaminase levels were linked to a greater probability of response, likely reflecting active parenchymal inflammation during an early phase, which may be more amenable to UDCA’s therapeutic effects. Importantly, both the ALP trajectory and treatment delay may serve as indicators of progressive biliary damage due to suboptimal management, underscoring the prognostic relevance of timely initiation of second-line therapies. A significant correlation between estimated treatment failure and histological indicators of disease severity, specifically ductular reaction, biliary metaplasia, and fibrosis extent, was demonstrated, thus underscoring the role of biliary injury severity, reflected by surrogate markers (ALP and bilirubin), as a predictor of responsiveness to choleretic treatment [141]. Despite the URS having been externally validated, showing high accuracy, this tool has not yet been included in routine clinical practice [141]. The simultaneous combination of the URS with LSM evaluation, using thresholds of <6.5 kPa and >11 kPa to, respectively, rule out and rule in advanced fibrosis, can further refine baseline risk stratification, categorizing patients into low, intermediate, and high-risk groups for future liver-related adverse outcomes [154,221].

More recently, machine learning (ML) models have been developed to predict treatment response in PBC, offering new avenues for precision medicine approaches. Specifically, in a large international population of PBC-patients, including a derivation cohort of 11,819 patients and a validation cohort of 1069 patients, ML-based clustering identified four distinct subgroups with unique clinical phenotypes and prognostic trajectories: patients with excellent long-term outcomes (cluster 1); individuals with worse prognosis, differing from cluster 1 primarily by albumin levels near the lower limit of normal (cluster 2); younger patients with pronounced cholestasis (cluster 3); advanced-stage cases with poor prognosis (cluster 4). Albumin dynamics emerged as a key prognostic marker, with an increase in albumin > 1.2× lower limit of normal (LLN) following UDCA therapy associated with significantly improved transplant-free survival [222].

Altogether, these findings underscore the growing relevance of predictive algorithms and biomarker-based stratification in guiding individualized therapeutic strategies in PBC. Integrating clinical scores with imaging and machine learning tools may enhance early identification of non-responders and optimize long-term outcomes.

## 4. Future Perspectives in Managing PBC: From One-Size-Fits-All to Tailored Approaches

The management of PBC is steadily moving towards a more personalized, dynamic, and stratified approach, aiming to overcome the traditional “one-size-fits-all” paradigm. This shift encompasses therapeutic decision-making, prognostication, monitoring strategies, and the timing of intervention.

**Figure 3:**
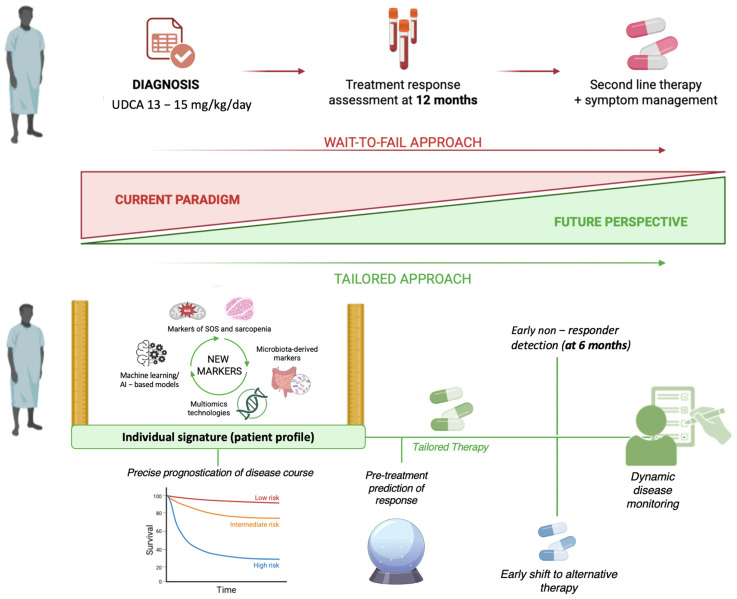
The current paradigm vs. the future perspective approaches in managing PBC patients. AI: artificial intelligence; SOS: Systemic oxidative stress.

Future research efforts are geared towards refining risk prediction models, identifying early non-responders (at 6 months), tailoring treatments, and predicting the response to both first-line and emerging additional treatments directly at the time of diagnosis. Improving current tools, such as their reliance on post-treatment biochemical data and limited applicability in second-line settings, will require the integration of advanced technologies, including ML, multi-omics platforms, and real-time biomarker profiling [43,154,222]. These innovations hold promise for constructing a more comprehensive and temporally sensitive patient profile, enabling earlier and more effective therapeutic interventions. The following sections report the most encouraging future perspectives driving the shift from the current strategies defining the wait-to-fail approach to the modern tools potentially contributing to a tailored management of PBC (Figure 3).

### 4.1. Time for New Markers: The Emerging Role of Multiomics in Predicting PBC Individual Evolution

To date, marker validation has primarily relied on conventional clinical, biochemical, and imaging parameters, which fail to fully capture disease heterogeneity and therapeutic response, whereas high-throughput omics technologies (encompassing genomics, epigenomics, transcriptomics, proteomics, metabolomics, and microbiomics) offer the opportunity to unravel disease complexity and generate molecular signatures more directly linked to pathogenetic mechanisms [43,154,222].

Circulating analytes, including microRNAs, cell-free DNA, and metabolite profiles, provide the basis for “liquid biopsy” approaches that may allow dynamic, non-invasive assessment of inflammation, biliary damage, and fibrosis [43,154,222]. Transcriptomic studies have identified gene expression and microRNA patterns correlating with disease progression, while proteomic and metabolomic analyses have revealed chemokine alterations (e.g., CCL20, CXCL11) associated with biliary epithelial cell senescence and non-response to UDCA, supporting their potential as mechanistic biomarkers in clinical trials [43,154,222].

Moving forward, the integration of validated biomarkers into predictive disease models requires large, longitudinal cohorts supported by standardized biobanking and data management systems.

Artificial intelligence and ML approaches will be instrumental to analyze such complex datasets, as shown by recent applications that stratified PBC patients into prognostically distinct biochemical clusters [43,154,222].

In parallel, polygenic risk scores and multiomics integration may eventually predict disease susceptibility and outcomes even before clinical onset, while advances in single-cell omics and patient-derived organoids promise to refine personalized disease modelling and accelerate drug discovery [43,154,222]. Although this represents a significant challenge, considering the cost and logistics of large-scale studies, data storage, privacy issues, and the need for rigorous validation, the convergence of omics technologies, computational tools, and collaborative infrastructures holds the potential to transform clinical practice, enabling precision prognostication, treatment tailoring, and ultimately interceptive medicine in PBC.

### 4.2. Microbiota-Derived Markers Composing Signatures to Predict Individual PBC Evolution

Emerging evidence underscores the pivotal role of gut microbiota alterations and metabolites as potential biomarkers and mechanistic mediators in PBC [223,224,225,226]. Consistently, PBC patients exhibit reduced gut microbial diversity and significant compositional shifts, including enrichment of potentially pathogenic genera such as *Veillonella*, *Streptococcus*, *Klebsiella*, *Clostridium*, *Lactobacillus*, and *Bifidobacterium*, alongside depletion of beneficial taxa like *Faecalibacterium*, *Oscillospira*, *Ruminococcaceae*, and *Christensenellaceae* [224,227]. Notably, faecal microbial profiles differ across fibrosis stages: advanced fibrosis is associated with reduced richness and elevated *Weisella* abundance, accompanied by altered short-chain fatty acid (SCFA) levels, such as increased acetate, suggesting a link between microbiota-derived metabolites and disease severity [225].

Recently, ML-based models have identified signature taxa, including decreased *Faecalibacterium* and *Gemmiger*, and increased *Veillonella* and *Streptococcus*, that accurately distinguish PBC patients progressing to cirrhosis from those who don’t (AUC ≈ 0.90) [228]. Moreover, baseline microbial community structures also emerged as a potential predictor of therapeutic response to UDCA: patients with a “Clostridia-low” microbiome subtype exhibit a twofold higher rate of UDCA non-response, along with diminished anaerobic fermentation and reduced production of bioactive metabolites such as tryptophan derivatives and secondary bile acids [229].

Interestingly, meta-transcriptomic and metabolomic profiling revealed that non-responders display decreased abundance of *Gemmiger* and *Prevotella*, elevated *Ruminococcus* and *Clostridium*, and disrupted bile acid-related microbial functions, implicating shifts in the enterohepatic circulation and pro-inflammatory immune signalling [230]. Mendelian randomization analyses further point to potential causal links between specific microbial taxa, such as Deltaproteobacteria (e.g., Desulfovibrionaceae), and reduced PBC risk, possibly related to bile salt hydrolase activity and modulation of intestinal barrier integrity, whereas increased abundance of taxa like Lachnospiraceae_UCG_004 may elevate PBC risk through their influence on secondary bile acid accumulation [231].

Altogether, these findings suggest that gut microbiota-derived markers, including specific bacterial taxa, functional signatures, and their metabolic outputs (e.g., SCFAs and bile acids), hold promise as non-invasive biomarkers for diagnostic, prognostic, and therapeutic stratification in PBC.

### 4.3. Systemic Oxidative Stress Markers: PBC Hepatic Progression and Extra-Hepatic Manifestations

Systemic oxidative stress (SOS) is increasingly recognized as a key factor in PBC, not only correlating with disease progression but also influencing muscle metabolism and itch pathways [40,232]. Recent evidence has demonstrated a correlation, direct for Reactive Oxygen Metabolites (dROMs) and inverse for Biological Antioxidant Potential (BAP), with liver fibrosis and steatosis, assessed by LSM and controlled attenuation parameter (CAP) [40,232]. Specifically, dROMs quantify circulating hydroperoxides as markers of oxidative burden, while BAP reflects the systemic antioxidant capacity [40,232]. In PBC patients, a progressive increase in dROMs (F0–F2 vs. F3: *p*: 0.0008; F3 vs. F4: *p*: 0.04) and a corresponding decline in BAP levels (F0–F2 vs. F3: *p*: 0.0007; F3 vs. F4: *p*: 0.04) according to the worsening of liver fibrosis were observed, reinforcing their potential as non-invasive indicators of disease severity [40,232]. Clarifying the pathogenetic mechanisms linking SOS to PBC represents a significant future research direction, potentially opening the doors to new markers of disease severity, such as dROMs and BAP.

Importantly, recent data revealed that SOS is associated with reduced muscle mass and increased pruritus intensity in PBC, in contrast to CLDs of other etiology, suggesting a disease-specific oxidative phenotype [232].

These findings indicate a mechanistic link between oxidative imbalance and muscle metabolism dysfunction, possibly mediated by mitochondrial stress and inflammatory signaling [232]. Moreover, vitamin D deficiency, frequently observed in PBC due to cholestasis-induced malabsorption, appears to exacerbate SOS and may contribute to both sarcopenia and pruritus via impaired antioxidant defense and neuromodulator pathways [232].

Taken together, the identification of systemic oxidative stress markers complements the expanding spectrum of omics- and microbiota-derived signatures. Beyond their mechanistic relevance, these markers collectively highlight the need to translate biomarker discovery into actionable tools for clinical decision-making, paving the way for their integration into personalized management strategies in PBC.

### 4.4. Clinical and Translational Implications of Emerging Biomarkers

The incorporation of novel biomarker signatures into clinical practice has the potential to refine decision-making in PBC management substantially, moving the field closer to precision hepatology. Beyond their mechanistic relevance, these markers may directly influence several critical aspects of patient care [233].

On one side, risk stratification at diagnosis could be enhanced by integrating omics-derived and microbiota-based profiles, allowing clinicians to identify patients at risk of rapid disease progression or non-response to UDCA earlier than conventional biochemical indices permit. Such stratification would support the timely initiation of second-line therapies, thereby reducing the reliance on the current “wait-to-fail” approach [47].

On the other hand, dynamic monitoring of disease activity through liquid biopsy markers—including circulating microRNAs, cell-free DNA, and metabolite signatures—offers the possibility of non-invasive, real-time assessment of therapeutic efficacy. This would enable clinicians to adjust treatment regimens proactively, minimizing unnecessary exposure to ineffective therapies and improving long-term outcomes [233].

Moreover, individualized treatment allocation may be informed by multiomics or microbiota-derived signatures, which could predict responsiveness to emerging agents such as FXR agonists, fibrates, or immunomodulatory therapies. By tailoring therapy to molecular and microbial profiles, clinicians could optimize drug selection, reduce adverse effects, and improve cost-effectiveness in clinical practice [234].

Furthermore, prognostic refinement could be achieved by incorporating systemic oxidative stress markers such as dROMs and BAP into existing risk models. These markers not only correlate with hepatic fibrosis but also anticipate extra-hepatic complications, including sarcopenia and pruritus, thereby broadening the scope of prognostication beyond liver-related endpoints [40,232,233].

Finally, the integration of biomarker signatures into clinical algorithms will require robust validation in large, longitudinal cohorts, harmonization of biobanking and data management infrastructures, and the application of advanced computational tools such as machine learning [235]. These steps are essential to ensure reproducibility, regulatory acceptance, and eventual incorporation into guidelines [235].

Collectively, these advances promise to transform biomarker discovery into actionable clinical tools, bridging mechanistic insights with precision medicine strategies in PBC. By enabling earlier intervention, dynamic monitoring, and individualized therapy, biomarker-driven approaches may ultimately improve survival, quality of life, and healthcare resource utilization in patients with PBC.

### 4.5. Body Composition Parameters and Liver Density as Emerging Prognostic Tools in PBC

As previously mentioned, emerging evidence highlights sarcopenia as a relatively common, still underexplored complication in PBC, independently associated with poor prognosis and potential implications on QoL and therapeutic response [40,236].

Compared to other CLDs etiologies, in PBC, sarcopenia exhibits a higher prevalence even in initial (F0-F2) fibrosis stages [(PBC 57.10% vs. Metabolic dysfunction-associated Steatotic Liver Disease (MASLD) 30.76%, Hepatitis B virus (HBV) 22.60%, Hepatitis C virus (HCV) 20.70%; *p* < 0.0001)], and it progressively worsens with the disease stage progression [40,236]. Moreover, in a retrospective cohort of 174 PBC patients, male sex (OR: 9.152) and LSM ≥ 12.8 kPa (OR: 4.539) were identified as independent risk factors for sarcopenia [236]. Importantly, sarcopenia emerged as a significant prognostic indicator associated with a markedly increased risk of LREs occurrence (HR: 4.058) in Cox proportional hazards models [236].

Recently, a retrospective study by Wang et al. evaluated body composition parameters and liver density in patients with PBC using computerized tomography (CT) imaging to explore their prognostic value [237]. In this research, sarcopenia and myosteatosis were defined using the third lumbar vertebra (L3) skeletal muscle index (SMI) and skeletal muscle density (SMD) obtained from CT imaging [237].

Interestingly, sarcopenia, myosteatosis, and sarcopenic obesity were common findings, although none showed a significant association with UDCA biochemical response or liver-related mortality. In contrast, mean liver density emerged as an independent predictor of both therapeutic response and liver-related death [237].

Conclusively, these results highlight the assessment of sarcopenia and mean liver density as novel prognostic imaging-based tools showing superior reliability compared with traditional body composition indices, supporting their potential role in risk stratification in PBC. In this sense, future research should investigate the impact of muscle impairment on QoL in PBC patients, especially considering the demonstrated correlation between muscle quantity and SOS/pruritus severity, simultaneously burdening QoL.

## 5. Conclusions

PBC remains a complex autoimmune cholestatic liver disease with variable progression and persistent therapeutic challenges. Although UDCA continues to represent first-line therapy, a growing subset of patients exhibits suboptimal response or intolerance, highlighting the need for alternative strategies. The emergence of second-line agents, such as PPAR agonists, and therapies targeting symptom burden (e.g., pruritus, fatigue) is expanding the treatment landscape.

Parallel advances in prognostic modelling and biomarker development are essential to identify non-responders early, guide treatment escalation, and refine long-term risk stratification. The integration of multi-omics platforms, ML, and high-throughput biomarker discovery holds promise for transforming disease management from one-size-fits-all to tailored approaches.

In the era of Precision Medicine, future priorities include shifting clinical trials toward validated long-term endpoints, overcoming recruitment barriers inherent to this rare disease and indolent course, and standardizing novel biomarkers.

## Figures and Tables

**Table 1:** Drugs approved and/or under investigation in Primary Biliary Cholangitis.

Drug/Agent	Target/Class	Mechanism of Action	Key Trials/Evidence	Clinical Status
UDCA	Hydrophilic bile acid	Replaces toxic bile acids; anti-cholestatic, cytoprotective, immunomodulatory	Multiple RCTs; Global PBC Study Group (n = 3902): ↑ transplant-free survival	First-line standard of care
Budesonide	Corticosteroid	Anti-inflammatory; high hepatic first-pass metabolism	RCTs: improved biochemistry; no histological benefit; contraindicated in cirrhosis	Off-label; limited to non-cirrhotic overlap
OCA	FXR agonist	Suppresses bile acid synthesis; ↑ bile flow	POISE (NCT03505723): ↑ biochemical response; COBALT (NCT02308111): failed on hard endpoints; EMA withdrawal (2024)	Withdrawn in EU; restricted in US
Bezafibrate	Pan-PPAR agonist (α, β/δ, γ)	↓ bile acid synthesis; anti-inflammatory, antifibrotic	BEZURSO (NCT01654731): 67% ALP normalization; real-world data: ↓ mortality/transplant risk	Off-label; not approved for PBC
Fenofibrate	PPAR-α agonist	↓ CYP7A1 activity; ↑ bile acid detoxification; anti-inflammatory	Open-label studies: biochemical improvement; safety concerns in advanced disease	Off-label; (cautions in decompensated PBC)
Elafibranor	Dual PPAR-α/δ agonist	Anti-inflammatory, antifibrotic; modulates bile acid metabolism	ELATIVE (Phase III) (NCT04526665): 51% composite response; FDA accelerated approval (2024)	Approved (US); second-line
Seladelpar	Selective PPAR-δ agonist	Anti-inflammatory; choleretic; ↓ IL-31 (pruritus mediator)	RESPONSE (Phase III) (NCT04620733): 62% response; ↓ pruritus; FDA accelerated approval (2024)	Approved (US); second-line
Saroglitazar	Dual PPAR-α/γ agonist	↓ bile acid synthesis; lipid modulation	Phase II/III: ~50% ALP reduction; safety concerns (ALT ↑); ongoing trials	Investigational
Linerixibat	IBAT inhibitor	Blocks ileal bile acid reabsorption; ↓ pruritus	GLISTEN (NCT04950127) & GLIMMER (NCT04603937) (Phase III): ↓ WI-NRS, improved sleep and QoL	Investigational
Golexanolone	GABA-A R modulator	Neurosteroid; targets central fatigue pathways	Phase 1b/2: under evaluation for fatigue in PBC	Investigational

UDCA: Ursodeoxycholic acid; OCA: obeticholic acid; FXR: Farnesoid X receptor; RCT: randomized control trial; PBC: Primary Biliary Cholangitis; PPAR: Peroxisome Proliferator-Activated Receptor; US: United States; EU: Europe; ALT: alanine aminotransferase; ALP: Alkaline phosphatase; QoL: Quality of life; IBAT: ileal bile acid transporter; GABA-A R: Gamma-Aminobutyric Acid-A receptor; FDA: Food and Drug Administration; EMA: European Medicines Agency; POISE: PBC OCA International Study of Efficacy; NCT: clinical trial number; COBALT: Confirmatory Trial of Obeticholic Acid in Primary Biliary Cholangitis; BEZURSO: BEZafibrate in combination with URSOdeoxycholic acid in primary biliary cholangitis. CYP7A1: Cytochrome P450 Family 7 Subfamily A Member 1; ELATIVE: Study of elafibranor in patients with primary biliary cholangitis; IL: Interleukin; RESPONSE: Response to Seladelpar in Subjects With Primary Biliary Cholangitis; GLISTEN: Global Linerixibat Itch Study of Efficacy and Safety in Primary Biliary Cholangitis; GLIMMER: clinical study on Linerixibat (an investigational drug) for treating itching associated with Primary Biliary Cholangitis; WI-NRS: Worst Itching Intensity Numerical Rating Scal; ↓: reduction; ↑: increase.

**Table 2:** Predictive Models for therapeutic response in patients with Primary Biliary Cholangitis.

Model	Type	Assessment Timepoint	Key Variables	Primary Endpoint	Performance/Notes
Paris I	Binary	12 months	ALP < 3 × ULN, AST < 2 × ULN, bilirubin < 1 mg/dL	Transplant-free survival	AUROC 0.81–0.82; HR for LREs: 4.22
Paris II	Binary	12 months	ALP ≤ 1.5 × ULN, AST ≤ 1.5 × ULN, normal bilirubin	LREs	HR for LREs: 4.47
Barcelona	Binary	12 months	ALP decrease > 40% or normalization	LREs	HR: 1.95
Rotterdam	Binary	12 months	Albumin, bilirubin	LT or death; LREs	HR: 4.16 (LT/death); 2.98 (LREs)
Toronto	Binary	24 months	ALP < 1.67 × ULN	Histologic progression	HR for LREs: 3.13
Early ALP (6 months)	Binary	6 months	ALP > 1.9 × ULN	Predicts POISE-defined non-response at 1 year	Identifies ~67% of non-responders early
GLOBE Score	Continuous	12 months	Age, ALP, bilirubin, albumin, platelets	LT-free survival (3–15 years)	AUROC: 0.83–0.94; HR for LREs: 3.03–5.05
UK-PBC Score	Continuous	12 months	ALP, bilirubin, transaminases, albumin, platelets	LT or liver-related death (5–15 years)	AUROC: 0.79–0.95; HR for LREs: 3.39

ALP = Alkaline phosphatase; AST = Aspartate aminotransferase; ULN = Upper limit of normal; LREs = Liver-related events; LT = Liver transplantation; HR = Hazard ratio; AUROC = Area under receiver operating characteristic curve; POISE: PBC OCA International Study of Efficacy; UK-PBC: United Kingdom-Primary Biliary Cholangitis; GLOBE: Global Primary Biliary Cholangitis Study Group

## Data Availability

No new data were created or analyzed in this study. Data sharing is not applicable to this article.

## Abbreviations

The following abbreviations are used in this manuscript:

ACA	Anti-centromere antibodies
AIH	Autoimmune hepatitis
ALBI	Albumin–Bilirubin score
ALP	Alkaline phosphatase
ALT	Alanine aminotransferase
AMA	Antimitochondrial antibodies
AMA-M2	Antimitochondrial antibodies subtype M2
ANA	Antinuclear antibodies
AAR	AST/ALT ratio
APRI	AST-to-platelet ratio index
AST	Aspartate aminotransferase
BAP	Biological Antioxidant Potential
BMI	Body Mass Index
CAP	Controlled attenuation parameter
CSPH	Clinically significant portal hypertension
CTP	Child–Turcotte–Pugh score
CT	Computed tomography
ELF	Enhanced Liver Fibrosis score
EMA	European Medicines Agency
ESLD	End-stage liver disease
FDA	U.S. Food and Drug Administration
FXR	Farnesoid X receptor
GGT	Gamma-glutamyltransferase
GWAS	Genome-wide association studies
HCC	Hepatocellular carcinoma
HRQoL	Health-related quality of life
HR	Hazard ratio
HVPG	Hepatic venous pressure gradient
IBAT	Ileal bile acid transporter
IgG	Immunoglobulin G
IgM	Immunoglobulin M
KLHL12	Kelch-like 12
LREs	Liver-related events
LSM	Liver stiffness measurement
MASLD	Metabolic dysfunction-associated steatotic liver disease
MELD	Model for End-stage Liver Disease
miR-	MicroRNA
ML	Machine learning
MRE	Magnetic resonance elastography
MRCP	Magnetic resonance cholangiopancreatography
MRS	Mayo Risk Score
NITs	Non-invasive tools
NRF2	Nuclear factor erythroid 2–related factor 2
OCA	Obeticholic acid
PBC	Primary biliary cholangitis
PDC-E2	Pyruvate dehydrogenase complex E2 subunit
PH	Portal hypertension
PPAR	Peroxisome proliferator-activated receptor
QoL	Quality of life
RPR	Red blood cell distribution width to platelet ratio
SCFA	Short-chain fatty acid
SMD	Skeletal muscle density
SMI	Skeletal muscle index
SOS	Systemic oxidative stress
SSM	Spleen stiffness measurement
TE	Transient elastography
TGF-β1	Transforming growth factor beta 1
ULN	Upper limit of normal
URS	UDCA Response Score
UDCA	Ursodeoxycholic acid
UK-PBC	United Kingdom Primary Biliary Cholangitis
